# The Role of Symmetric Stem Cell Divisions in Tissue Homeostasis

**DOI:** 10.1371/journal.pcbi.1004629

**Published:** 2015-12-23

**Authors:** Jienian Yang, Maksim V. Plikus, Natalia L. Komarova

**Affiliations:** 1 Department of Mathematics, University of California, Irvine, Irvine, California, United States of America; 2 Department of Developmental and Cell Biology, Sue and Bill Gross Stem Cell Research Center and Center for Complex Biological Systems, University of California, Irvine, Irvine, California, United States of America; 3 Department of Ecology and Evolutionary Biology, University of California, Irvine, Irvine, California, United States of America; Max-Planck-Institute for Evolutionary Biology, GERMANY

## Abstract

Successful maintenance of cellular lineages critically depends on the fate decision dynamics of stem cells (SCs) upon division. There are three possible strategies with respect to SC fate decision symmetry: (a) asymmetric mode, when each and every SC division produces one SC and one non-SC progeny; (b) symmetric mode, when 50% of all divisions produce two SCs and another 50%—two non-SC progeny; (c) mixed mode, when both the asymmetric and two types of symmetric SC divisions co-exist and are partitioned so that long-term net balance of the lineage output stays constant. Theoretically, either of these strategies can achieve lineage homeostasis. However, it remains unclear which strategy(s) are more advantageous and under what specific circumstances, and what minimal control mechanisms are required to operate them. Here we used stochastic modeling to analyze and quantify the ability of different types of divisions to maintain long-term lineage homeostasis, in the context of different control networks. Using the example of a two-component lineage, consisting of SCs and one type of non-SC progeny, we show that its tight homeostatic control is not necessarily associated with purely asymmetric divisions. Through stochastic analysis and simulations we show that asymmetric divisions can either stabilize or destabilize the lineage system, depending on the underlying control network. We further apply our computational model to biological observations in the context of a two-component lineage of mouse epidermis, where autonomous lineage control has been proposed and notable regional differences, in terms of symmetric division ratio, have been noted—higher in thickened epidermis of the paw skin as compared to ear and tail skin. By using our model we propose a possible explanation for the regional differences in epidermal lineage control strategies. We demonstrate how symmetric divisions can work to stabilize paw epidermis lineage, which experiences high level of micro-injuries and a lack of hair follicles as a back-up source of SCs.

## Author Summary

Stem cells have long been associated with their ability to divide asymmetrically, when one daughter cell retains stem cell properties of the parent cell, while the other daughter cell becomes more mature and loses its stemness. Recent findings, however, point at the existence of an alternative, symmetric division strategy for stem cells in mammalian tissues. Here we ask the question: what engineering design principles might be responsible for optimization of stem cell division strategies? Although simple intuition may suggest that asymmetric divisions are better suited for stable maintenance of cell population numbers, our analysis shows that asymmetric divisions can also destabilize the system, depending on the particular “wiring” of the control loops that govern cellular fate decision making. We apply our theory to one particular unresolved question in mouse epidermis studies—why symmetric division percentage in paw epidermis is twice as high as that in ear and tail? The answer may be related to the fact that paw epidermis lacks hair follicles (a source of stem cells available in other types of skin), and it is also more injury-prone: symmetric divisions help stabilize skin in face of physical stresses from running, digging, grooming, and fighting—things that mice do with their paws. Thus, our theory offers quantitative explanations for the observed designs in stem cell lineages.

## Introduction

All cells within the body organize into distinct phylogenetic lineages. At the end of each lineage are the non-dividing, terminally differentiated cells. Usually these cells, such as neurons, adipocytes or muscle fibers, are highly specialized and endow tissues with their respective functions. The origin of all differentiated cells can be traced back to their progenitors, the so-called stem cells (SCs) [1, 2]. Typically, tissue-specific SCs are long lasting and self-renewing (i.e. at least 50% of SC progeny remain as SCs) [2, 3]. They also maintain high proliferative potential and assure lifelong lineage survival both under physiological steady-state conditions, and upon lineage depletion after injury or disease. These SC properties are vividly demonstrated by the experiments when the entire tissues are restored from just one grafted SC. For instance, new functional prostate tissue can reform following transplantation of one prostate SC under kidney capsule [4]. Similarly, transplantation of a single hematopoietic SC can reconstitute the entire bone marrow in lethally irradiated mice that would otherwise die from the inability to make new blood [5–7]. Another example is scarring alopecia, the type of baldness caused by the autoimmune attack on hair SCs—once SCs are lost, hairs can never grow again [8, 9].

Successful maintenance and repair of cellular lineages critically depends on the fate decision dynamics of SCs upon division. Long-term steady-state maintenance of lineages requires that only 50% of all SCs progenies remain as SCs, and even slight shift in fate outcomes over time can lead to lineage exhaustion or uncontrolled expansion. For example, in the hair follicle, melanocyte SCs are more susceptible to exhaustion compared to epithelial SCs; therefore, commonly hair graying occurs faster than hair loss [10–12]. On the other hand, uncontrolled lineage expansion occurs upon myelodysplastic syndrome, a type of blood malignancy when mutated hematopoietic SCs increase their self-renewal rate to more than 50%. Over time, mutated SCs outcompete normal SCs, and accumulation of defective progeny cells leads to the loss of blood function and results in acute myeloid leukemia, a life-threatening complication of the myelodysplastic syndrome [13–15].

**Fig 1:**
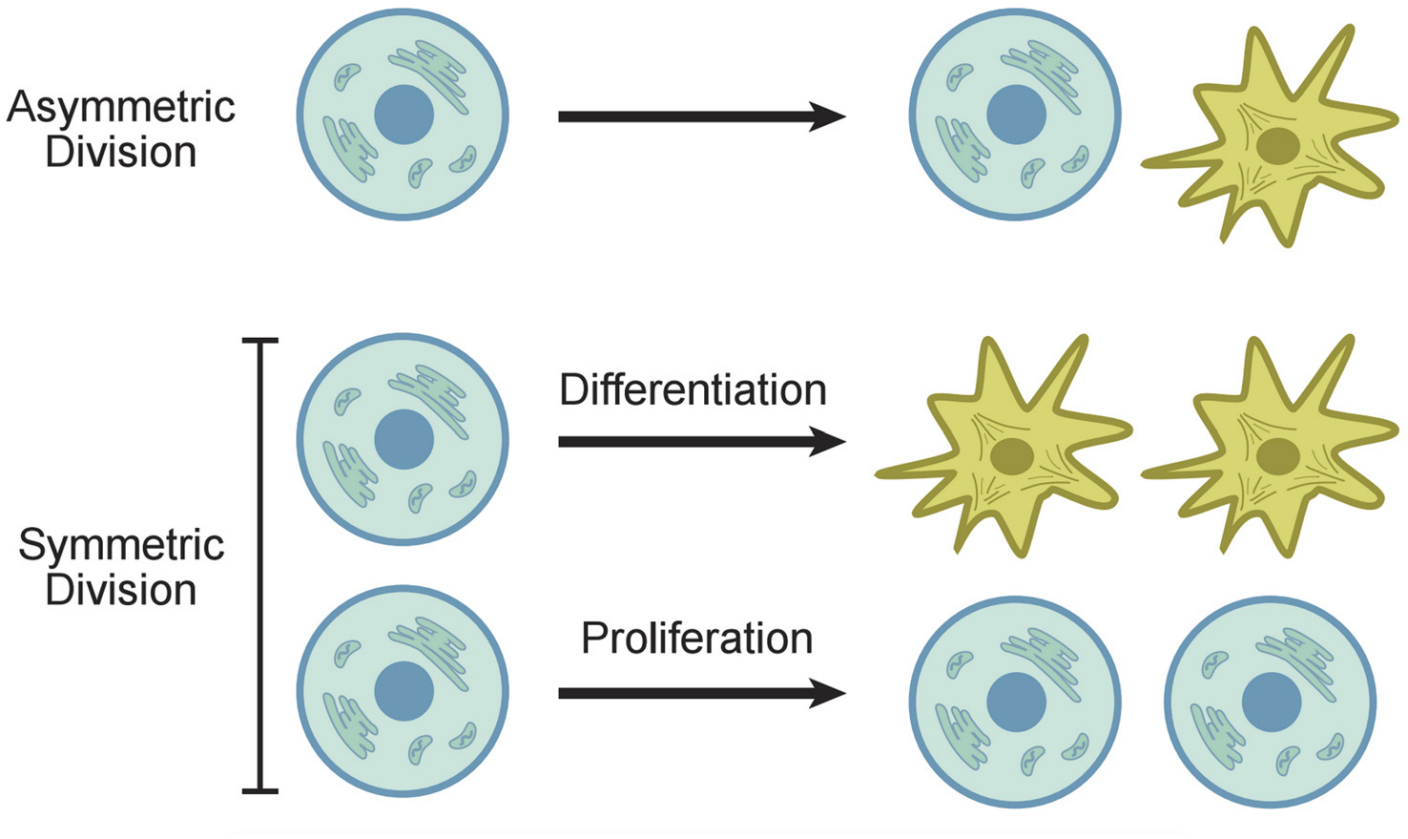
Symmetric and asymmetric SC divisions. In the asymmetric division model, a SC produces one differentiated cell and one SC. In the symmetric division model, a SC produces two differentiated cells or two SCs.

From these examples it is evident that tight control of SC fate decision dynamics is of paramount importance. In principle, steady-state maintenance of SCs can be achieved with three strategies [16], see Fig 1:

a) Asymmetric mode, when each and every SC division produces one SC and one non-SC progeny [17–22];
b) Symmetric mode, when 50% of all divisions produce two SCs and another 50%—two non-SC progeny. In this case SC expansion is precisely balanced by SC exhaustion, and the long-term net balance of SCs and their lineages remains unchanged [16, 23, 24];
c) Mixed mode, when both the asymmetric and two types of symmetric SC divisions co-exist and are partitioned so that long-term net balance of the lineage output stays constant. As in (b), stability critically depends on the ratio of symmetric divisions: SC generating events should be precisely counterbalanced by SC exhaustion.

Assuming that individual cell division decisions are stochastic, at the tissue level, modes (b) and (c) result in neutral clone competition phenomenon, when some SC clones expand, some contract, while others stay constant [16, 25–28]. Theoretically, either one of the strategies (a-c) can achieve lineage homeostasis. However, it remains unclear which strategies are more advantageous and under what specific circumstances, and what minimal control mechanisms are required to operate them.

From the control point of view, SC fate can be decided either intrinsically or extrinsically [16]. Intrinsic control implies that the fate of daughter cells is determined by the signals present within the mother SC. Generally, this strategy is used only in specialized circumstances, for example during initial stages of embryonic development in C. elegans or in Drosophila neuroblasts [29, 30].

Extrinsic control strategy, when the fate of daughter cells is determined at the time of SC division by the instructive environmental signals is more prevalent in adult tissues and organs. Depending on the source of the signal, autonomous and non-autonomous control mechanisms are recognized [16]. Non-autonomous mode is common in complex, highly regenerative tissues, such as hair follicles and bone marrow, and requires presence of the so-called niche cells [31–35]. These, often specialized cells of mesenchymal origin, generate the complex signaling micro-environment that regulates multiple aspects of lineage behavior both in time and in space, including but not limited to: (i) SC quiescence vs. proliferative activation [36–38], (ii) symmetric vs. asymmetric fate of daughter cells [21, 23, 39], (iii) progressive specification and differentiation of non-SC progenies [40–42], (iv) switch between homeostatic vs. damage-response lineage behaviors [10, 43, 44].

For instance, skin hair follicle SCs activate cyclically. During the rest phase of the cycle, specialized mesenchymal niche supplies hair SCs with strongly inhibitory signals, keeping them quiescent. However, at the onset of the new hair growth phase, the same niche cells start to supply SCs with strongly activating signals. Furthermore, upon skin wounding, hair follicle SCs are guided to enter an “emergency” mode and transiently change their default fate: rather than sending progenies toward hair root, they send them upwards into the newly forming wound epidermis [43, 45–47]. Generally, anatomic and signaling complexity of SC niches allows for spatial-temporal separation of SC activities and a plethora of lineage behaviors depending on the specific tissue, organ, as well as organismal needs. At the same time, such regulatory complexity makes it difficult to study the key homeostatic property of SCs—the fate decision control upon division.

In addition to non-autonomous, niche-based SC control, some tissues display largely autonomous regulation. The latter mechanism implies regulation of SCs by their own daughter cells, both of SC and non-SC kind. Autonomous strategy is commonly seen in tissues with relatively simple microanatomy, such as stratified epidermis in mammalian skin [25–28, 48–50]. Skin epidermis is vertically stratified and is arranged in successive layers of epithelial cells. SCs occupy lowermost basal layer, while their non-SC progeny, including terminally differentiated cells, move into the upper suprabasal layers. Depending on the anatomical location, epidermal thickness can range from just 2–3 layers, like in the back skin of mice, to several dozens, like the in mouse’s paw skin [51]. Multiple genetic tools for lineage tracing and signaling perturbations make mouse epidermis a particularly attractive experimental system for studying mechanisms of autonomous lineage control. Furthermore, because epidermal SCs switch their proliferative behavior in ontogenesis and upon wound healing, it enables studying aspects of plasticity of lineage control networks.

In the present paper we address the questions of SC division symmetry by means of mathematical modeling. Our approach is based on that developed in [52, 53], and it contributes to the large theoretical literature on SC dynamics, see e.g. theoretical work of [54–57], and a review in [58]. Some of the important areas of mathematical modeling in the context of SCs include discrete and continuous models in the context of carcinogenesis [59–71]; modeling of SC in the hematopoietic system [72–76]; deterministic modeling of two-, three-, and multi-compartmental systems under various regulation functions [77–81]; stochastic modeling of SC systems and the analysis of fluctuations [82–87].

The focus of the present study is to investigate how different division types contribute to lineage homeostasis/turnover. We provide analysis that allows to quantify the ability of two types of divisions (symmetric and asymmetric) to maintain homeostasis. What SC division strategy is better at maintaining the nearly constant population size? Quoting [16], “Asymmetric divisions are a key mechanism to ensure tissue homeostasis. In normal stem and progenitor cells, asymmetric cell division balances proliferation and self-renewal with cell-cycle exit and differentiation.” At the intuitive level, it appears that asymmetric SC divisions should be associated with a more robust homeostatic maintenance. It can be argued that purely asymmetric SC divisions do not change the total number of SCs and therefore ensure the maintenance of a constant cell population, see e.g. [18]. It turns out however that tight homeostatic maintenance of the lineage (including differentiated cells) is not necessarily associated with purely asymmetric divisions. In this paper we show that asymmetric divisions can either stabilize or destabilize the lineage system, depending on the underlying control network.

Once we establish the relationship between symmetry of cellular fate and lineage stability, we apply our computational model to biological observations in the context of mouse epidermal SCs and autonomous lineage control. It has been observed [25–28] that the proportion of symmetric divisions is higher in mouse paw epidermis compared to that of the tail and ear. By using our model we propose an explanation for this phenomenon.

## Results

**Fig 2:**
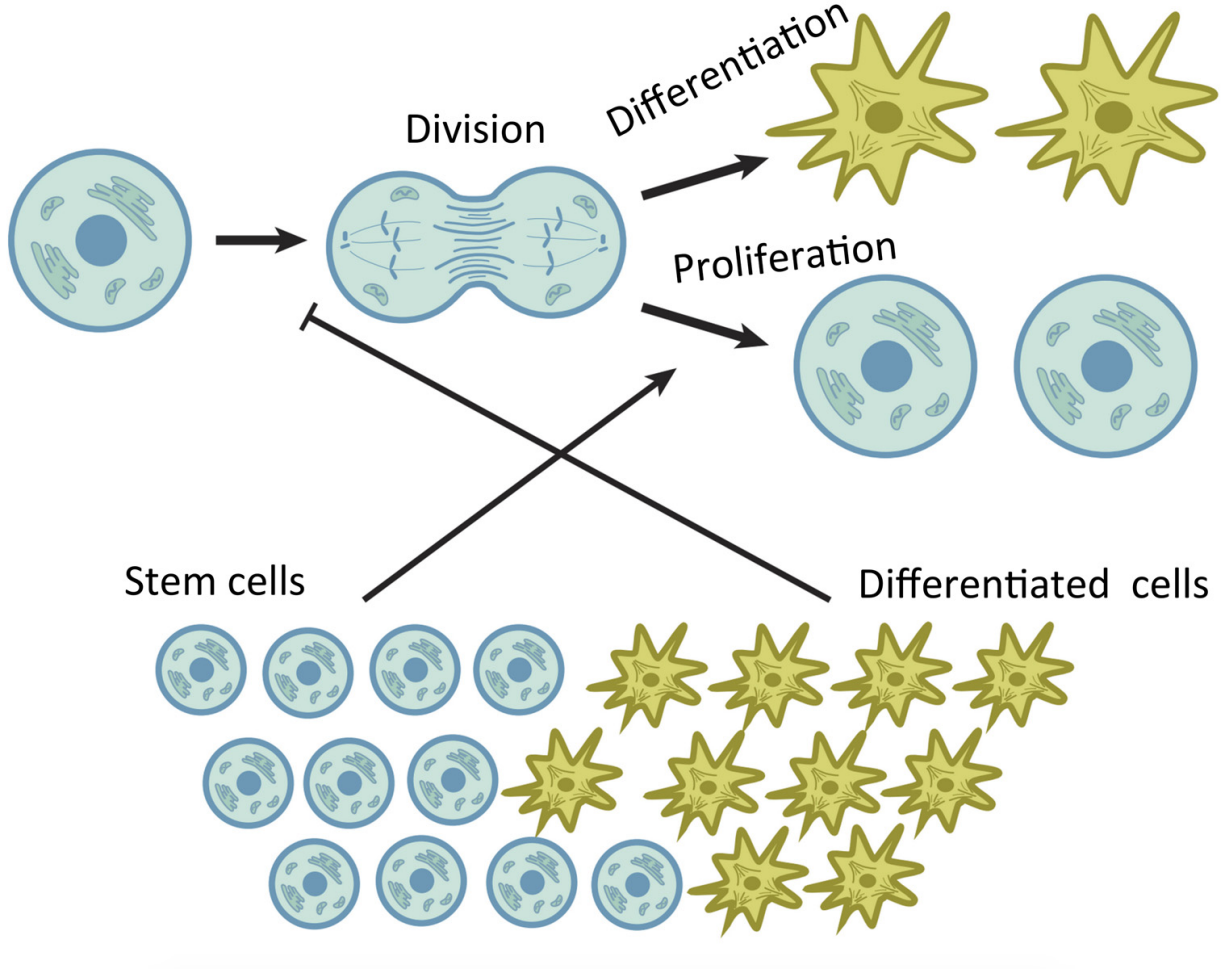
An example of endogenous control loops regulating SC decisions with the all symmetric division mode. Division events are negatively regulated by daughter cells and differentiation decisions are positively regulated by SCs.

We study a stochastic model with various control loops that distinct cell populations impose on the prevalence of different processes. For example, consider the simplest lineage, which only consists of SCs and one type of daughter cells. We postulate that such a system consists of two compartments, that of SCs and the differentiated cells. We further assume that the rate of SC divisions, and also the probability of differentiation/proliferation (see Fig 1), are controlled by chemical factors (such as morphogenetic growth factors) secreted by cells of different compartments, as well as exogenous factors coming from outside of the lineage (such as distinct niche cells). In Fig 2, the endogenous controls are illustrated by using a simple example of symmetric divisions of SCs. An individual decision tree of a SCs is depicted schematically. It consists of the decision to undergo a division, followed by the decision about the nature of this division (that is, whether daughter cells will maintain SC fate or undergo differentiation). In the example in Fig 2, the probability to divide is limited by the population of daughter cells. If there are too many of them, this will reduce the chances of further divisions. In the same system, the probability of differentiation is influenced by the number of SCs. The more SCs there are, the more likely they will be to differentiate, thus reducing the total SC number. We refer to this system (which in the case of Fig 2 consists of only two controls) as a control network. In the above scenario, one control is positive, and the other is negative.

It is possible to construct many other control networks that consist of different numbers of positive and/or negative controls. In [52] we have shown that the control network in Fig 2 (along with many other networks) is compatible with stable maintenance of a constant cell population size. The resulting system of cells is characterized by a stochastic behavior, where the numbers of stem and daughter cells fluctuate around certain mean values. The size of these fluctuations is an important characteristic of a biological system. If these fluctuations are too large (compared to the means) then the population is running a danger of going extinct, which will be a catastrophic outcome for a biological system. The smaller the fluctuations, the more robust is the system and the tighter is homeostatic maintenance. We are interested in the general question of design: what features of control improve the robustness of the system in the sense described above.

In Fig 2, only symmetric divisions are considered. At the next level of complexity we also consider the possibility of asymmetric divisions. Thus, we can assume that SCs can divide both symmetrically and asymmetrically, with a given relative probability. Here we study how the balance between symmetric and asymmetric SC divisions can change the robustness properties of the lineage. What percentage of divisions should be symmetric to minimize fluctuations for tighter homeostatic control?

Qualitative intuitive reasoning suggests that asymmetric divisions must be associated with the highest level of stability of SC lineages. In the case of asymmetric divisions, the number of SCs does not change, because every time a SC divides, it replaces itself with exactly one SC, and also produces a differentiated cell. Therefore, it might seem that under fully asymmetric divisions, as long as the production of differentiated cells is balanced on average by their deaths, the system will be stable. It turns out however (see the Methods section) that depending on the exact control loops acting in the system of SCs and non-SC daughter cells, asymmetric divisions might either increase or decrease lineage size fluctuations. This is what we demonstrate next.

### The role of division symmetry in stable homeostasis: The case of minimal control systems

Let us suppose that the lineage consists of two types of cells (two compartments), SCs and daughter (differentiated) cells. Let us denote by *I* and *J* the current number of stem and daughter cells, respectively. The processes of division (including differentiation/proliferation decisions) and death are dictated by probabilities and rates defined in Table 1(a). Next, we need to quantify the control loops that exist in a given system.

**Table 1:** Notations used in the models.

**(a) Processes**	
*L*_*I*, *J*_	Division rate of SCs
*S*_*I*, *J*_	Probability that the division is symmetric
*P*_*I*, *J*_	Probability that a symmetric division is a differentiation event
*D*_*I*, *J*_	Death rate of differentiated cells
**(b) Controls**	
*q*_*x*_(*q*_*y*_)	Partial derivative of *L*_*I*, *J*_ − *D*_*I*, *J*_ with respect to the argument *I* (*J*), evaluated at the equilibrium.
*p*_*x*_(*p*_*y*_)	Partial derivative of *P*_*I*, *J*_ with respect to the argument *I* (*J*), evaluated at the equilibrium.

(a) Definitions of rates and probabilities. Subscripts denote functional dependence on the cell populations *I* and *J*. (b) The four partial derivatives evaluated at the equilibrium comprise the four controls in a two-compartment system.

We assume that *L*_*I*, *J*_ = *L*(*ϵI*, *ϵJ*), *D*_*I*, *J*_ = *D*(*ϵI*, *ϵJ*), etc, where *ϵ* measures the strength of dependence of the probabilities and rates on the cell population numbers. It is convenient to introduce the continuous variables *x* = *ϵI*, *y* = *ϵJ*. To define the control network, we consider the partial derivatives of the rates and probabilities with respect to *x* and *y*, evaluated at the equilibrium. We will use the subscripts *x* and *y* to denote such partial derivatives, see Table 1(b). A two-compartment system is characterized by the following four derivatives: *p*_*x*_, *p*_*y*_, *q*_*x*_, and *q*_*y*_, which we call *controls*. To clarify the biological meaning of these parameters, consider the quantity *p*_*y*_. If it is nonzero, it means that the probability of SC differentiation is controlled by the differentiated cell population. Moreover, if *p*_*y*_ < 0, this means that the control is negative (the more differentiated cells in the system, the less likely the SCs are to differentiate); *p*_*y*_ > 0 means the existence of a positive control loop. The other three quantities can be interpreted in a similar manner.

**Fig 3:**
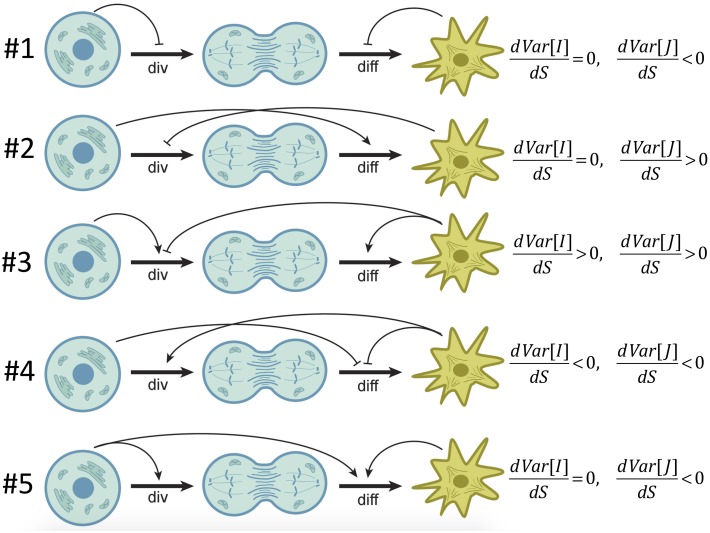
Classification of minimal control systems in two-compartment models. Symbol “div” refers to the rate of symmetric stem cell divisions (both proliferations and differentiations). Symbol “diff” refers to the probability of differentiation; the probability of proliferation is 1-Prob(diff). Models #1–2 are the two-control systems. Models #3–5 are three-control systems. Division and differentiation decisions can be positively or negatively controlled by the population sizes of SCs or differentiated cells, as indicated by arch-like arrows that originate at the relevant cell population and point toward the process that this population controls. The rightmost column indicates how cell number variances depend on the symmetry of divisions, as obtained from the analysis of the Methods Section.

It was shown in [52] that at least two of the four controls must be nonzero in order for the system to have a stable homeostatic equilibrium. Minimal control systems are defined as models with a restricted number of nonzero controls, and are presented in Fig 3. In the schematic, round cells and star-like cells represent stem and differentiated cells respectively. The first horizontal arrow in each diagram indicates the division decision, and the second horizontal arrow the differentiation decision. Arch-like positive and negative arrows depict the dependence of the two decisions on each population. For example, if a negative arrow originates at SCs and points at the divisions decision, this means that the divisions are negatively controlled by the SC numbers, *q*_*x*_ < 0 (see diagram #1 in Fig 3). It was shown in [52] that with two compartments, there are two distinct minimal control systems with two controls, and three systems with three controls (see also S1 Text).

The first two models (#1 and #2) in Fig 3 are the only two systems that can be stable in the presence of no more than two controls. The other three models (#3–5 in Fig 3) are the only three irreducible three-control systems, that is, they cannot be reduced to models #1 or #2 by setting one of the controls to zero. While from the point of view of stability, all five of the networks are possible, further biological considerations are required to identify which control network is relevant for a particular tissue. Some of those considerations may include the matching of various moments of compartment sizes with the observations, robust recovery dynamics, etc. In the particular case study considered in this paper (mouse epidermis) network #5 appears to be the most relevant, as explained below.

Next we demonstrate how by varying the proportion of symmetric vs asymmetric SC divisions, one can change homeostatic properties of the system in the context of models #1–5. We will focus on the analysis of variance of the cell populations. A relatively small variance indicates stable, robust homeostasis. A large variance increases the probability of extreme events, such as extinction or growing out of control. By using stochastic analysis (see the Methods Section) we can calculate the variance of the number of SCs, *Var*[*I*], and the variance in the number of differentiated cells, *Var*[*J*], as functions of the parameters. In particular, it is possible to determine how these quantities depend on the four controls (Table 1(b)) and the frequency of symmetric SC divisions, *S*. It turns out that in two out of five control systems in Fig 3, the variance increases with *S*. Namely, in systems #2 and #3, *Var*[*J*] increases with *S*, and in addition, in #3 *Var*[*I*] also increases with *S* (in #2, the variance of SC numbers is independent of the symmetry), see Eqs (33) and (34). Therefore, in these two control systems, purely asymmetric divisions are optimal from the viewpoint of minimizing fluctuations in cell numbers at homeostasis.

The opposite result is observed for systems #1, #4, and #5. There, purely symmetric divisions turn out to be the optimal choice. In those three systems, the variance of differentiated cell numbers is a decreasing function of *S*, and in addition, in #4, the variance of SC numbers is also a decreasing function of *S*, see Eqs (32), (35) and (36). In these three qualitatively different control networks, symmetric divisions are associated with the most stable homeostatic state. Next, we demonstrate this theoretical finding by numerical simulations.

### Application to two control systems

The results reported in the previous section hold for any functional forms of controls. Here we illustrate these findings by considering two specific examples. Some technical details about the simulation setup are provided in S1 Text. Recall that *ϵ* measures the strength of control of the various processes by the cell population, and *x* = *ϵI*, *y* = *ϵJ*; we further denote Δ = *q*_*x*_ *p*_*y*_ − *q*_*y*_ *p*_*x*_, and *B* = 2L_*_ S_*_(*p*_*x*_ − *p*_*y*_) − *q*_*y*_, where the partial derivatives with respect to *x* and *y* are defined in Table 1(b) and the star indicates that the quantity is evaluated at the equilibrium. The quantities Δ and *B* appear in the expressions for the variances (Methods). Throughout this section, we will assume *S*_*I*, *J*_ takes some constant value *c*, where 0 < *c* ≤ 1. Although *S*_*I*, *J*_ is not necessarily constant, its derivatives do not enter the stability conditions or expressions for population variances (as explained in the Methods section), and therefore we make the simplest assumption on this function. Below are two examples, where in order to illustrate the theory numerically, we chose some specific functional forms for the controls.

#### Model #3

Consider three-control model #3 from Fig 3, which is characterized by negative regulation of division (by differentiated cells) and positive regulation of division (by SCs) and differentiation (by differentiated cells). As an example of this kind of a model, we assign the following functional forms of the controls:

$$\begin{array}{ccc} L_{I,J} & = & L\left(\epsilon I,\epsilon J\right)=\frac{1-e^{-\epsilon I}}{1-e^{-\epsilon I}+\epsilon J},\;P_{I,J}=P\left(\epsilon I,\epsilon J\right)=1-e^{-3\epsilon J},\\ D_{I,J} & = & D\left(\epsilon I,\epsilon J\right)=1-L_{I,J},\;S_{I,J}=c. \end{array}$$ (1)

We therefore have *p*_*x*_ = 0, *p*_*y*_ = *ϵ*3*e* ^−3*y*^ > 0, *q*_*x*_ = *ϵ*2*ye* ^−*x*^ ⋅ (1 − *e* ^−*x*^ + *y*)^−2^ > 0, $q_{y}=\epsilon \frac{2e^{-x}-2}{{\left(1-e^{-x}+y\right)}^{2}}<0$. The steady state of the system can be obtained by solving *P*(*x*, *y*) = 1/2, *L*(*x*, *y*) = *D*(*x*, *y*) (system Eq (16) in Methods):

$$\begin{array}{c} i_{0}=-\frac{\log(1-\log2^{1/3})}{\epsilon },\;j_{0}=\frac{\log2}{3\epsilon }. \end{array}$$

By Eq (24), we can obtain the means and the variances of the system:

$$\begin{array}{ccc} E[I] & = & i_{0}, \end{array}$$ (2)

$$\begin{array}{ccc} E[J] & = & j_{0}, \end{array}$$ (3)

$$\begin{array}{ccc} Var[I] & = & \frac{2L_{*}S_{*}\Delta +q_{y}^{2}+8L_{*}^{2}S_{*}p_{y}^{2}}{4B\Delta }, \end{array}$$ (4)

$$\begin{array}{ccc} Var[J] & = & \frac{2L_{*}\left(2+S_{*}\right)\Delta +q_{x}^{2}}{4B\Delta }, \end{array}$$ (5)

where all the partial derivatives are evaluated at (*i*_0_, *j*_0_), and L_*_ = 1/2, Δ = *q*_*x*_ *p*_*y*_, *B* = −2L_*_ S_*_ *p*_*y*_ − *q*_*y*_.

**Fig 4:**
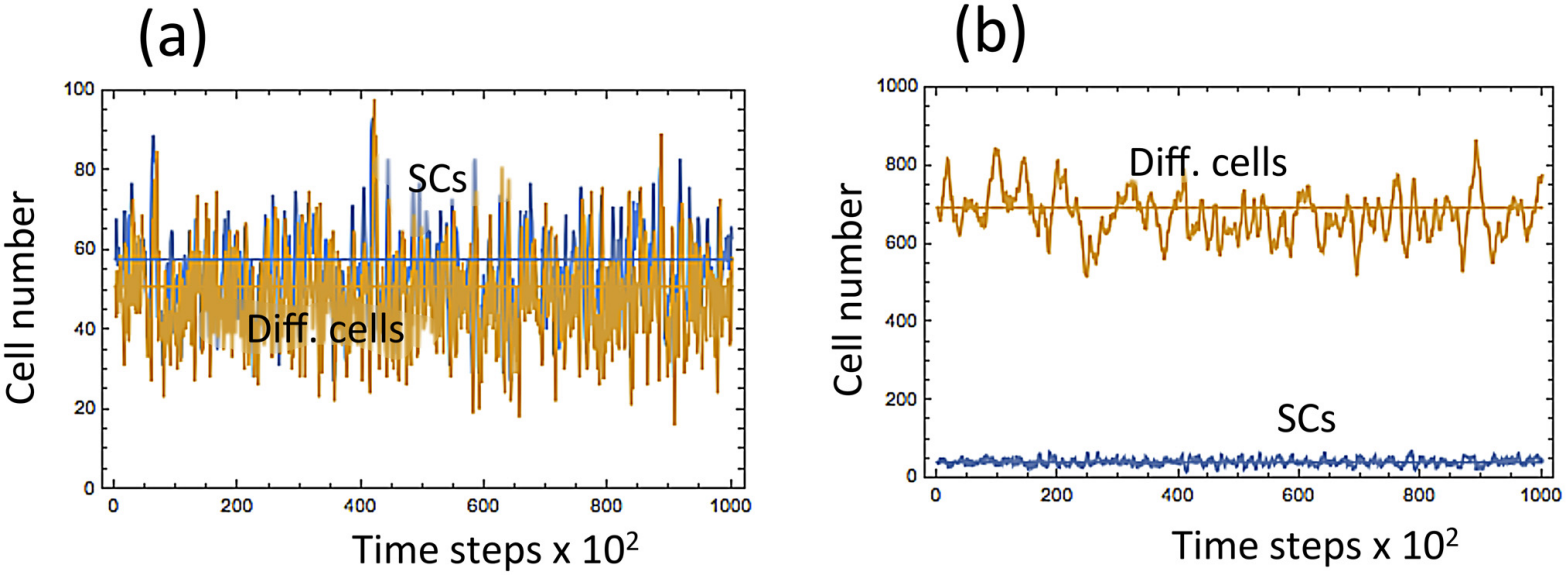
Typical numerical simulations of cell dynamics. (a) System Eq (1) with *ϵ* = 0.005 and S_*_ = *S* = 0.5; (b) system Eq (6) with *ϵ* = 0.005 and S_*_ = *S* = 0.8. Simulations are run for 2 ⋅ 10^5^ time steps.

For each fixed pair (*ϵ*, S_*_), we ran numerical simulations starting at the (rounded up) expected values of the cell population given above, and finishing either when the number of time-steps reached 2 ⋅ 10^5^, or if any of the cell types went extinct. We then computed the means and the variances of the cell population over the time-course of each simulation. A typical run for a particular parameter set is presented in Fig 4(a). In other simulations, both *ϵ* and S_*_ varied between 10^−3^ and 10^0^.

**Fig 5:**
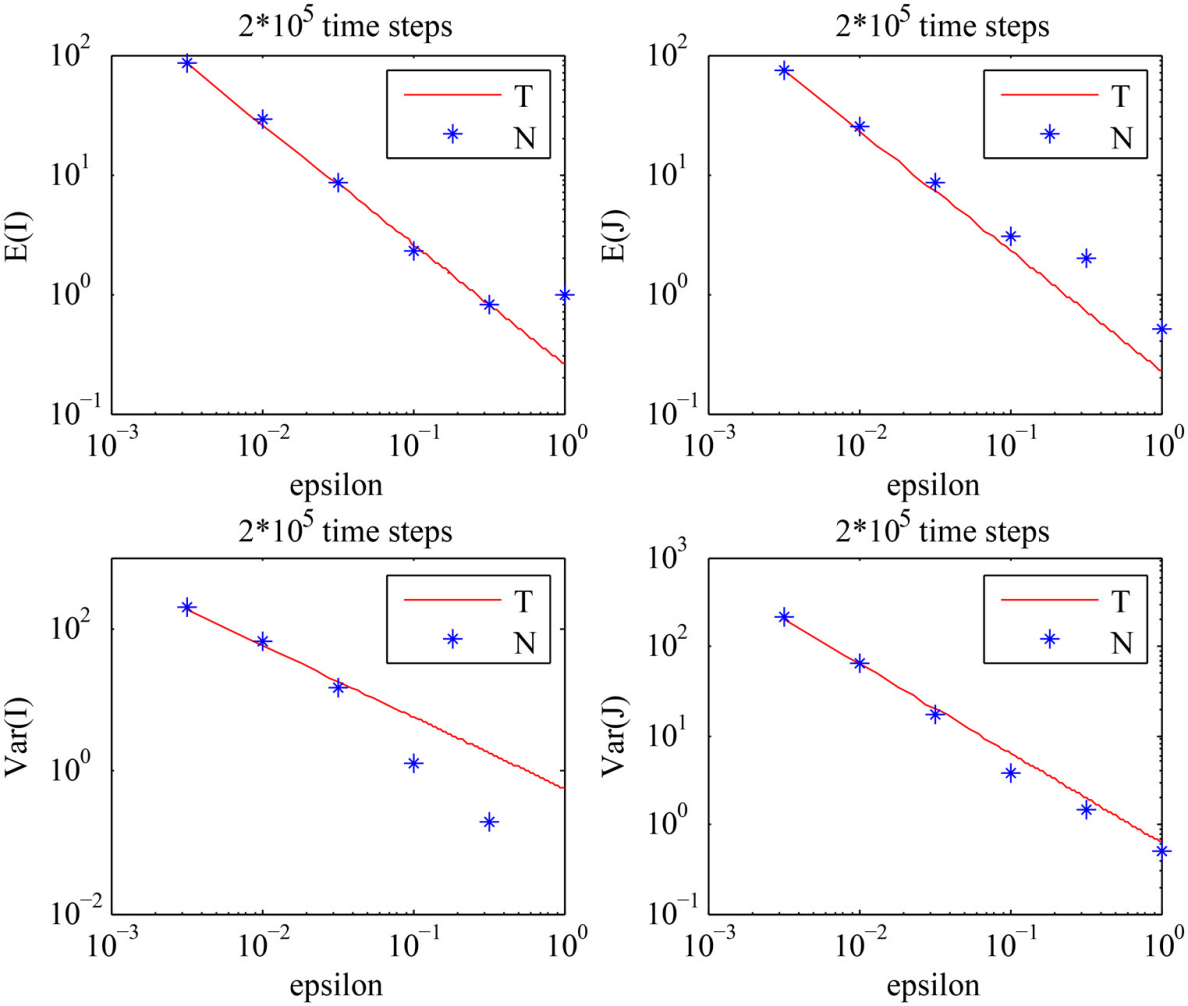
The behavior of the means and the variances of the cell population described by Eq (1). The analytical results given by Eqs (2–5) (solid line) are compared with the values obtained by numerical simulations (stars), for different values of *ϵ* with the fixed value of *S*: *S* = 0.5. The choice of *S* should satisfy: *S* = S_*_ < *S*_*c*_, where *S*_*c*_ is given by Eq (29). (‘T’) stands for the theoretical results, and (‘N’) stands for the numerical results.

**Fig 6:**
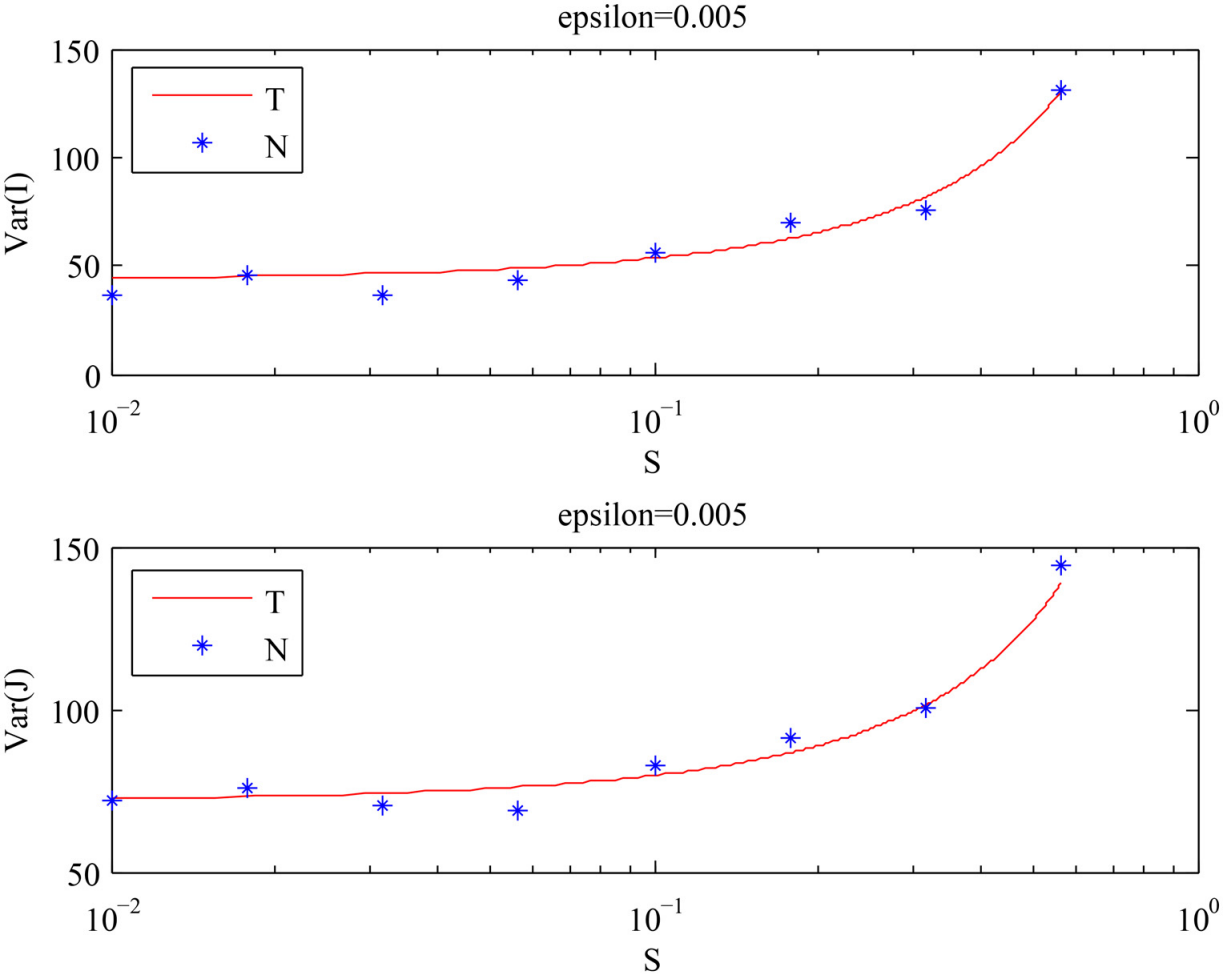
The behavior of the variances of the cell population described by Eq (1) with *ϵ* = 0.005, for different values of *S*. The analytical results given by Eqs (2–5) (solid line) are compared with the values obtained by numerical simulations (stars). (‘T’) stands for the theoretical results, and (‘N’) stands for the numerical results.

From Fig 5, we observe that the theoretical results for the means and the variances show a good agreement with the numerical results for smaller values of *ϵ*, which is what we expect. We further observe that the means and the variances of the cell population decrease as the value of *ϵ* increases, exactly as predicted by Eqs (2–5). From Fig 6, we can see that the variances of the cell population increase as the value of *S* increases, which is consistent with the analytical results given by Eq (34).

#### Model #5

As the second example, we consider three control model #5 in Fig 3, which is characterized by positive control of differentiation and division. We will assign the following equations to the probability and rate functions:

$$\begin{array}{ccc} L_{I,J} & = & L\left(\epsilon I,\epsilon J\right)=\frac{2\tanh(\epsilon I)}{2\tanh(\epsilon I)+0.4},\;P_{I,J}=P\left(\epsilon I,\epsilon J\right)=\tanh\left(\epsilon I+0.1\epsilon J\right),\\ D_{I,J} & = & D\left(\epsilon I,\epsilon J\right)=1-L_{I,J},\;S_{I,J}=c. \end{array}$$ (6)

A typical stochastic simulation of system Eq (6) for a particular parameter set is presented in Fig 4(b). To calculate the variances, we find *p*_*x*_ = *ϵ* sech^2^(*x* + 0.1*y*) > 0, *p*_*y*_ = *ϵ*0.1sech^2^(*x* + 0.1*y*) > 0, *q*_*x*_ = *ϵ*1.6sech^2^(*x*) ⋅ (2tanh(*x*) + 0.4)^−2^ > 0, *q*_*y*_ = 0, and hence *p*_*x*_ > *p*_*y*_ > 0. The steady state of the system is

$$\begin{array}{c} i_{0}=\frac{\log1.5}{2\epsilon },\;j_{0}=\frac{\log3-\log1.5}{0.2\epsilon }. \end{array}$$

By Eq (24), we can obtain the means and the variances of the system:

$$\begin{array}{ccc} E[I] & = & i_{0}, \end{array}$$ (7)

$$\begin{array}{ccc} E[J] & = & j_{0}, \end{array}$$ (8)

$$\begin{array}{ccc} Var[I] & = & \frac{2L_{*}S_{*}\Delta +8L_{*}^{2}S_{*}p_{y}^{2}}{4B\Delta }, \end{array}$$ (9)

$$\begin{array}{ccc} Var[J] & = & \frac{2L_{*}\left(2+S_{*}\right)\Delta +q_{x}^{2}+8L_{*}^{2}S_{*}p_{x}^{2}}{4B\Delta }, \end{array}$$ (10)

where all the partial derivatives are evaluated at (*i*_0_, *j*_0_), and L_*_ = 1/2, Δ = *q*_*x*_ *p*_*y*_, *B* = 2L_*_ S_*_(*p*_*x*_ − *p*_*y*_). Note that S_*_ in Eq (9) cancels out.

**Fig 7:**
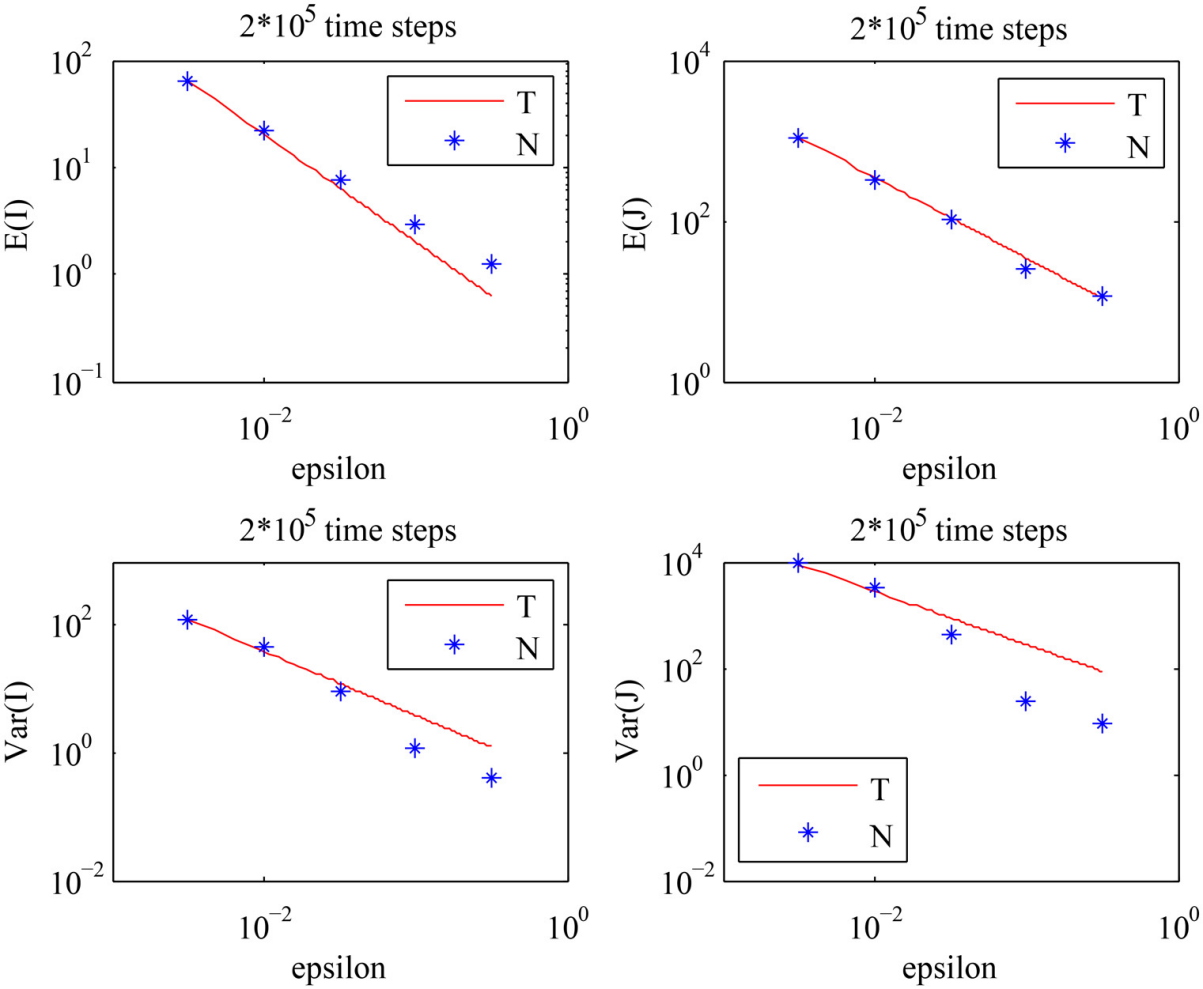
The behavior of the means and the variances of the cell population described by Eq (6). The analytical results given by Eqs (7–10) (solid line) are compared with the values obtained by numerical simulations (stars), for different values of *ϵ* with the fixed value of *S*: *S* = 0.5. The choice of *S* should satisfy: *S* > *S*_*c*_ = 0 in this case, where *S*_*c*_ is given by Eq (29). (‘T’) stands for the theoretical results, and (‘N’) stands for the numerical results.

**Fig 8:**
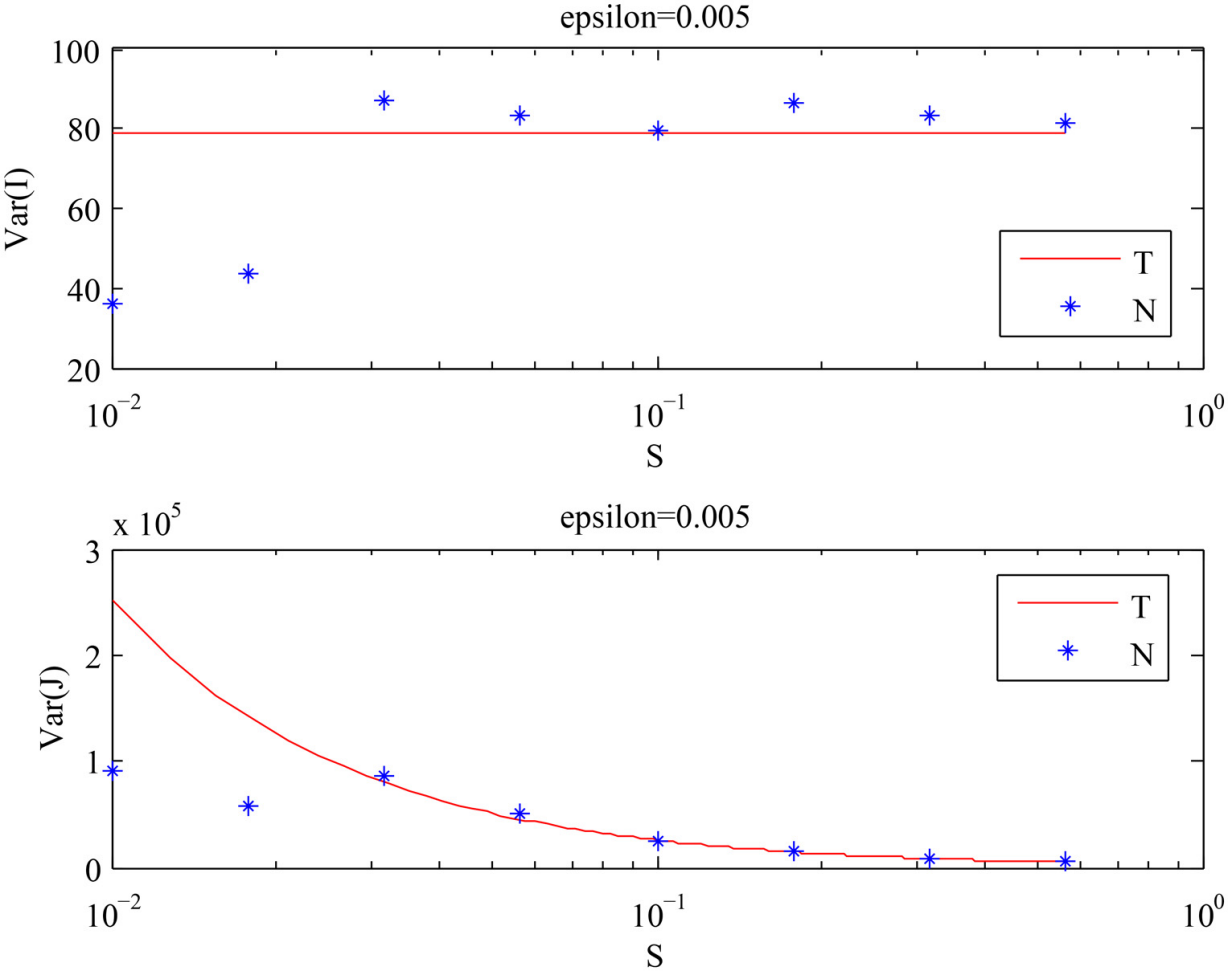
The behavior of the variances of the cell population described by Eq (6) with *ϵ* = 0.005 and time steps = 2 ⋅ 10^6^, for different values of *S*. The analytical results given by Eqs (7–10) (solid line) are compared with the values obtained by numerical simulations (stars). (‘T’) stands for the theoretical results, and (‘N’) stands for the numerical results.

**Fig 9:**
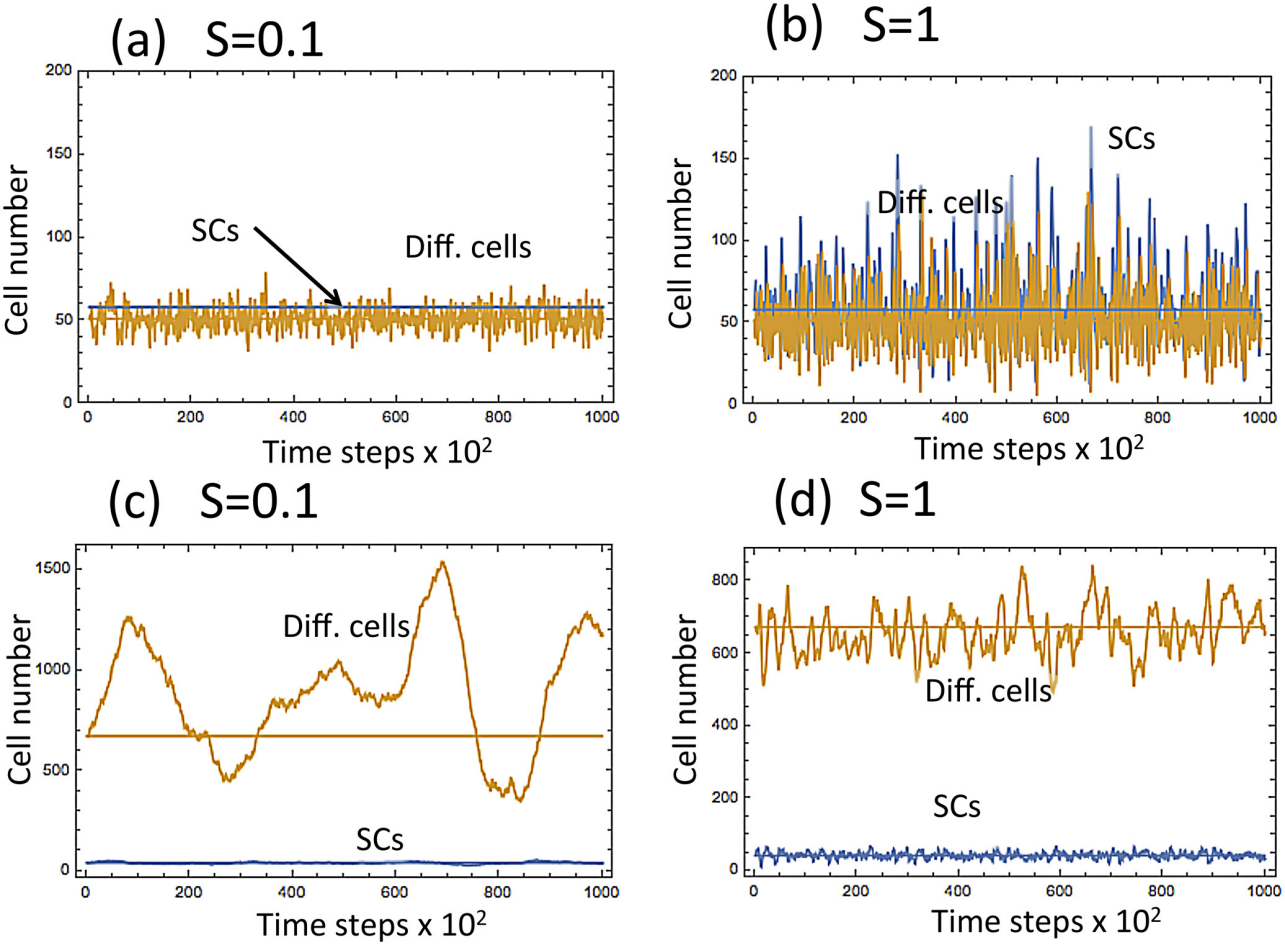
The behavior of the two systems described by Eqs (1) and (6) with *S* = 0.1 and *S* = 1. The top two diagrams, (a) and (b)m correspond to the first example, and the bottom two diagrams, (c) and (d), correspond to the second example. In Panels (a) and (c), *S* = 0.1 (mostly asymmetric divisions). In (b) and (d), *S* = 1 (symmetric divisions).

We used the same numerical scheme as in the previous example. As observed in Fig 7, the theoretical results are again in good agreement with the numerical results for smaller values of *ϵ*. The means and the variances of the cell population decrease as the value of *ϵ* increases, which is foretold by Eqs (7–10). From Fig 8, we observe that *Var*[*I*] stays approximately constant, whereas *Var*[*J*] decreases as *S* increases. The first two points in Fig 8 that appear to be inconsistent with the theory are explained by the analysis of the purely asymmetric divisions solution for smaller values of *S*, see S1 Text. The results of both numerical experiments are summarized in Fig 9, which shows that:

- Increasing the fraction of symmetric division destabilizes the system given by Eq (1);
- Increasing the fraction of asymmetric division destabilizes the system given by Eq (6).

### A case-study: Mouse epidermis

Mammalian epidermis develops through several distinct stages. In mice, one cell layer-thick epidermis first appears at embryonic day E8.5 [88–90]. Over the course of next few days and till day E13.5, primordial epidermal cells divide strictly symmetrically along the horizontal plane of the skin, and this contributes to the rapid expansion of epidermal surface in synchrony with rapid growth of the embryo body [89, 91].

Starting from E13.5, fully symmetric SC-generating strategy switches to a mixed mode, consisting of both symmetric and asymmetric divisions [48–50, 88]. Asymmetric divisions that generate one basal SC and one suprabasal non-SC comprise approximately 70% of all divisions in day E15.5 mouse embryos [50]. The 30% symmetric divisions at that time are likely necessary for epidermis to add more SCs and to grow in absolute area as embryo continues to enlarge.

What is the division strategy in adult epidermis that stopped expanding and reached its steady state? Several recent studies demonstrate that adult mouse epidermis is maintained via a mixed division mode, with basal SCs undergoing all three division types: asymmetric and two types of symmetric divisions. The support for the mixed mode is provided by the low-dose induction lineage tracing experiment. In this experiment, an inducible genetic system is used to randomly and permanently label few scattered basal epidermal SCs and all of their progenies. Labeling of just a few basal SCs assures that most of the marked SCs will be far from one another to prevent fusion of their progeny populations. Because over time, the total number of labeled clones decreased, while some of the remaining clones expanded in size, this supports symmetric divisions: loss of clones results from divisions generating two non-SCs, while expansion of clone sizes results from divisions generating two SCs [27, 28].

Interestingly, the exact ratio of division types appears to differ depending on the anatomical location. For example, in the mouse tail epidermis the ratio of asymmetric to SC + SC symmetric to non-SC + non-SC symmetric divisions is approximately 80%:10%:10% [26, 28] and in the mouse ear it is 78%:11%:11% [27]. However, in the footpad epidermis, it appears to change in favor of symmetric divisions—60%:20%:20% [25]. We would like to find an explanation of this increased fraction of symmetric SC divisions in the footpad compared to the ear/tail epidermis.

While little is known about the signaling aspects of SC fate determination in adult epidermis, the available published biological data point toward non-intrinsic mechanism. Mitotic spindle analysis indicates that, in contrast to embryonic epidermis, only 3% of basal SCs in adult mouse epidermis divide strictly vertically [28]. Moreover, daughter cell fate selection appears to depend on the dynamic signaling inputs generated in the basal layer: (i) short-range acting WNT ligands promote basal SC division, and (ii) long-range Dkk signal drives cell differentiation, i.e. non-SC identity [25, 92]. This type of autocrine/paracrine signaling from SCs closely matches our minimal control system #5, see Fig 3. Indeed in system #5, SCs exert positive control on their own division (matching the role of WNT ligands) as well as positive control on differentiation decision (matching the role of Dkk1). Also, control network #5 requires positive regulation of lineage differentiation by differentiated cells. Therefore, we speculate that other signaling events, in addition to WNT/Dkk1, are likely involved in regulating epidermal lineage homeostasis. Indeed, multiple signaling pathways, including Notch, TGF*β*, IKK, Ras/MAPK, PI3K and p63 have been implicated in regulation of epidermal proliferation and differentiation (reviewed in [93]). Of these, Notch signaling is of a particular interest. Notch signaling is active predominantly in suprabasal epidermal cells, where it drives their differentiation [94–97], matching third signaling event in the minimal control system #5.

What differences can account for the increase in symmetric divisions in the footpad epidermis? While to date, this issue has not been studied experimentally, the three most notable distinctions of the footpads from other body sites are:

1. Significantly increased epidermal thickness. Footpad epidermis in mice is nearly twenty layers-thick comparing to just several layers in other areas [51]. This difference however is only due to an increased thickness of suprabasal layer. The SC numbers are the same for the ear, the tail, and footpad.
2. Because of the functional differences in the epidermis, the footpad will experience an elevated level of micro-injuries to the tissue, resulting from abrasions and friction (paws are used for running, digging, grooming, fighting, etc.).
3. Lack of hair follicles. Footpads are distinctly hairless, and some populations of hair follicle SCs have been shown to contribute to epidermal maintenance in the back and tail skin [98, 99].

We will use mathematical modeling to propose an answer to the following question: Why does footpad epidermis have a larger proportion of symmetric divisions? We will explore the above three differences to see if any of them favors symmetric divisions, in the context of stable homeostatic tissue maintenance.

#### (i) Increased epidermal thickness

First we note that the necessity of more differentiated cell layers is not a sufficient explanation for the larger fraction of symmetric divisions in the footpad. During embryonic time, there is rapid expansion of the skin surface area. Thus, as skin surface is growing, symmetric divisions work to expand the population of epidermal SCs. This is consistent with the observation of high levels of symmetric divisions in early mouse embryo, between days E8.5 and E13.5. In adult animals, however, epidermal surface area remains relatively constant, and there is no need to expand the size of the epidermal SC population. As noted earlier, in principle, an equilibrium cell population can be maintained by purely symmetric, purely asymmetric, or mixed divisions, and therefore an increased thickness of the epidermis per se cannot explain the necessity for an increased frequency of symmetric SC divisions.

Below we will use a number of models to investigate the role of micro-injuries and hair follicles in the cellular dynamics in the context of epidermal SC division symmetries. As discussed above, we will base our models on variants of control network #5 in Fig 3, because it is the most realistic network rooted in the current knowledge about the signaling regulation of epidermal lineage.

#### (ii) Increased level of micro- injuries

**Fig 10:**
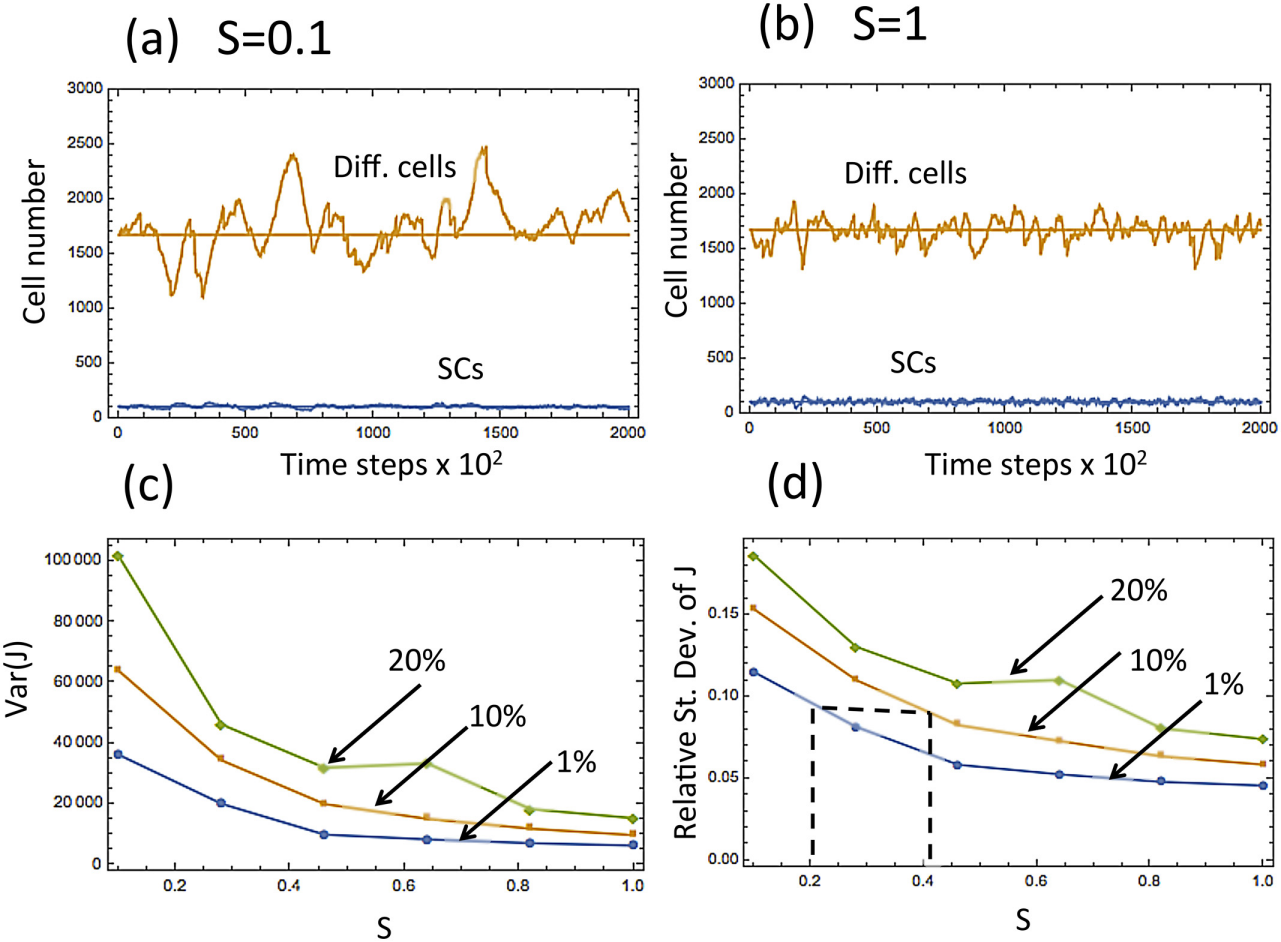
Modeling micro-injuries by introducing a certain percent (1%, or 10%, or 20%) decrease in the number of differentiated cells in 0.01% of temporal updates (randomly chosen). In (a,b) the numbers of SCs and differentiated cells is plotted as functions of time, for a typical run. In (a), most divisions are asymmetric (*S* = 0.1), and in (b), all divisions are symmetric (*S* = 1). (c,d) The variance and the relative standard deviation of the number of differentiated cells as a function of the percentage of symmetric divisions. The percent decrease of the number of differentiated cells is marked above each line. Eq (6) with *ϵ* = 0.002 was used.

In the first model of micro- injuries, we will use the control system already analyzed (Eq (6)), and assume that at a small fraction of updates, a fixed fraction of the total number of differentiated cells is removed from the system. Fig 10 demonstrates the results of the simulations with varying values of *S*_*I*, *J*_ = *c*, the frequency of symmetric divisions. We assumed that in 0.01% of the updates (chosen randomly), the number of differentiated cells is reduced by a certain fraction (the values 1%, 10%, and 20% were used). In Fig 10(a) we set *c* = 0.1, which corresponds to mostly asymmetric divisions, and in Fig 10(b) we have purely symmetric divisions (*c* = 1). As before, in the presence of this random noise, the system with predominantly asymmetric divisions is characterized by significantly larger fluctuations of the differentiated cell population. In Fig 10(c) and 10(d) we plot the variance and the relative standard deviation of the population level of differentiated cells, as a function of the percentage of symmetric divisions (relative standard deviation is defined as $\sqrt{Var(J)}/E\left(J\right)$). One can see that they are decreasing functions of the symmetry of divisions.

Our analysis suggests the following possible explanation for an increased number of symmetric divisions in the mouse footpad. Let us suppose that in the tail and in the ear (low-stress epidermis) a certain regulatory loop with 20% symmetric divisions is able to maintain a tolerably low level of tissue variance (see Fig 10(d), where this is marked by the leftmost vertical dashed line). In the footpad, due to an increased level of stress from micro-injuries, the same mechanism would lead to a much higher level of population size variation. In order to reduce this level to the tolerable bounds, the tissue can increase the percentage of symmetric divisions in the SCs, which will decrease variance (and possibly also increase the thickness of the epidermis). If we assume that the micro- injuries in the footpad are modeled by the 10% curve in Fig 10(d), then symmetric divisions at the level *S* ≈ 0.4 would yield the same level of fluctuations as in the low-stress epidermis (see the rightmost vertical dashed line). This ability of symmetric divisions to counter-balance the fluctuations resulting from micro-injuries may be a possible reason for an increased percentage of symmetric SC divisions in the footpad epidermis.

It is important to note that the specific values of the level of noise in the system depend on all the numerical parameter values and the functional forms of the controls. The values *S* = 0.2 and *S* = 0.4 shown in Fig 10, which coincide with the experimentally observed values for the ear/tail and the footpad, are chosen for illustration purposes. Changing parameter values will lead to different numbers, but the general trend remains robust. In the environment characterized by an increased level of noise, an increase in the percentage of symmetric divisions will improve the stability of the SC lineage.

In S1 Text, we present several alternative models of micro- injuries. In some of them, instead of removing cells from the system, we increase the death rate of the differentiated cells in a fixed fraction of updates. In other variants of the model we also include a reduction in the number of SCs (we note however that since physical injury to epidermis is coming from the outside, i.e. directed at the differentiated cells layer, it is more plausible that differentiated cells, rather than SCs (that sit deeper) are damaged directly). In a given model, several combinations of parameter values have been tested, and the basic trend is always the same: under an increased level of injuries, higher fraction of symmetric divisions can increase stability of the tissue in the sense of decreasing the relative standard deviation of differentiated cell population.

#### (iii) Lack of hair follicles

**Fig 11:**
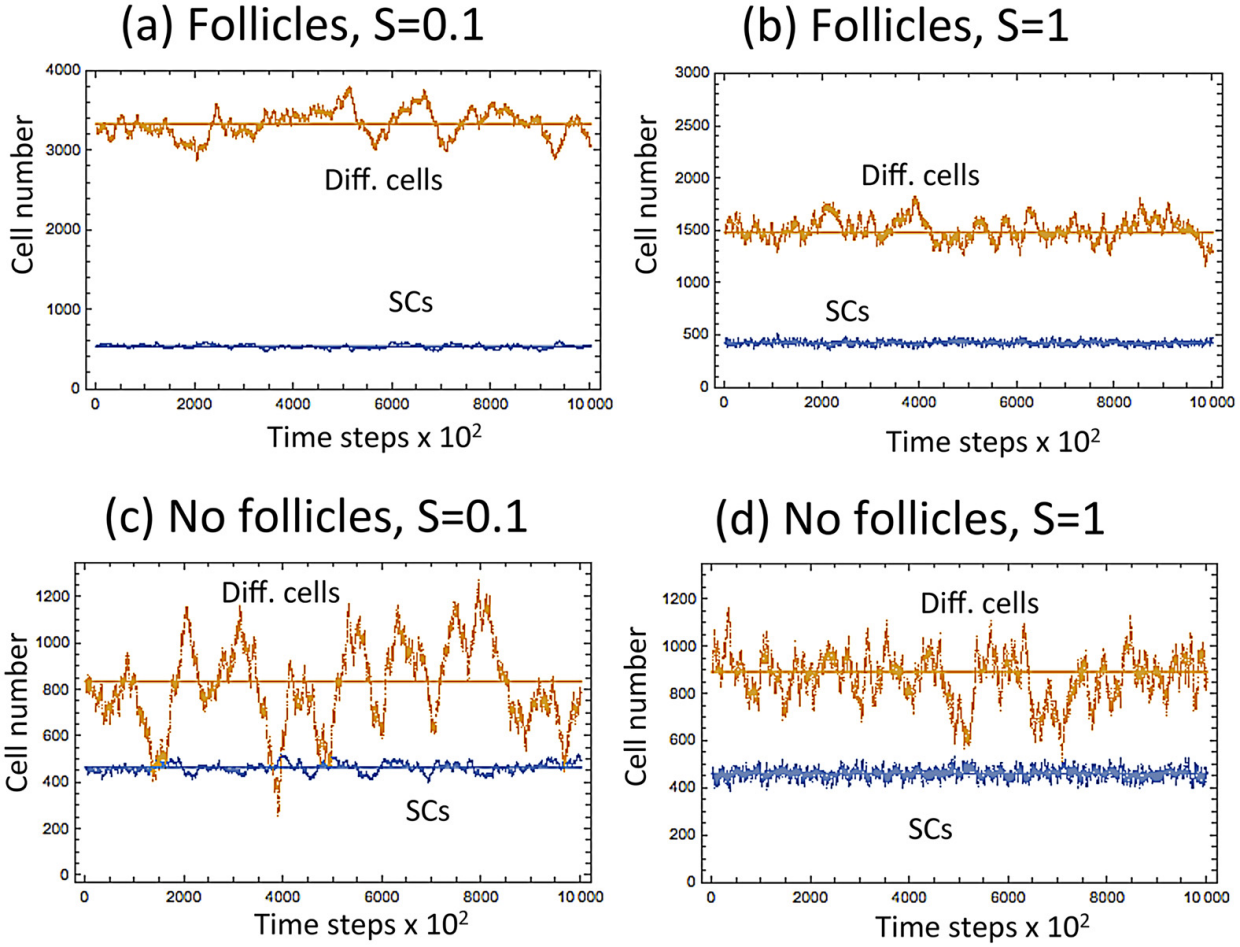
Modeling the possible role of hair follicles in epidermis turnover. The numbers of differentiated and SCs are plotted as functions of time, for a typical run. (a) *S* = 0.1, in the presence of follicles; (b) *S* = 1, in the presence of follicles; (c) *S* = 0.1, in the absence of follicles; (d) *S* = 1, in the absence of follicles. Eqs (11 and 12) were used with parameters *h* = 0.3, *ϵ* = 0.05.

**Fig 12:**
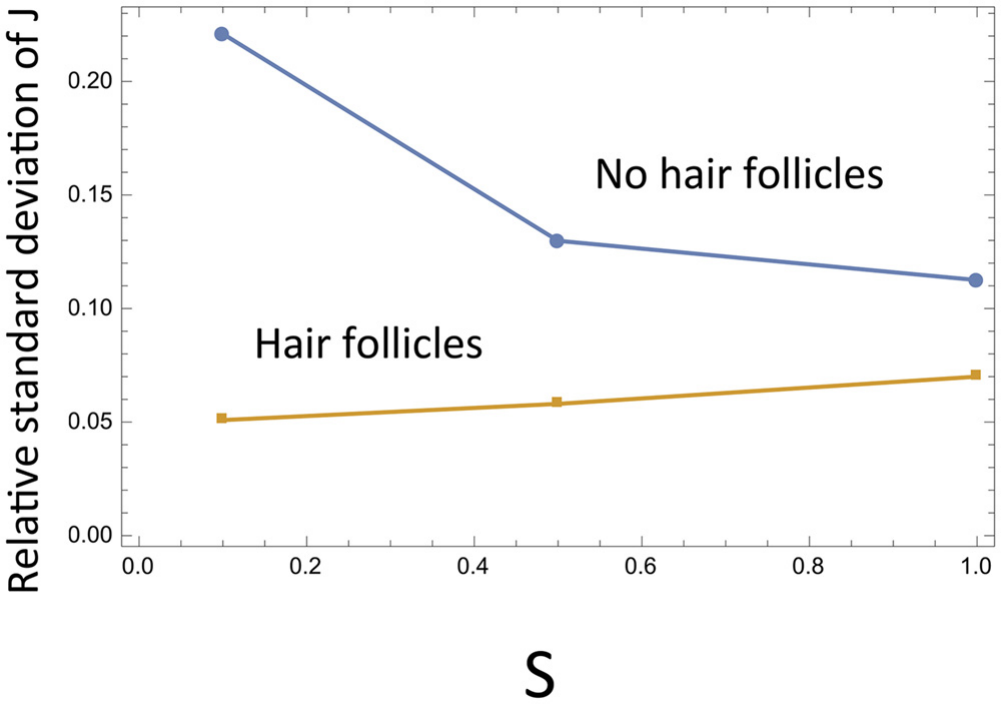
The role of hair follicles in epidermis turnover. The relative standard deviation of the number of differentiated cells ($\sqrt{Var(J)}/E\left(J\right)$) is plotted against the fraction of symmetric divisions, in the absence and in the presence of hair follicles. Parameters are as in Fig 11.

The lack of hair follicles in the footpad can also contribute to the increased symmetric divisions. To simulate the impact of hair follicles we assumed the following functional forms of the various processes:

$$\begin{array}{ccc} L_{I,J} & = & \frac{0.9\tanh(\epsilon I)}{2\tanh(\epsilon I)+0.4},\;P_{I,J}=\tanh\left(\epsilon I+0.1\epsilon J\right), \end{array}$$ (11)

$$\begin{array}{ccc} D_{I,J} & = & h+0.01\epsilon J,\;S_{I,J}=c,\;E=\frac{0.02}{1+\epsilon I}, \end{array}$$ (12)

where the last quantity, *E*, denotes the exogenous source of SCs in the system, and is assumed to be a decreasing function of SCs present in the population (see S1 Text for more details and also a general analysis independent of specific functional forms). The role of hair follicles in tissue stability under different frequencies of symmetric SC divisions is illustrated in Fig 11. In the presence of hair follicles (the top two graphs in Fig 11), we can see that lower levels of symmetric divisions (Fig 11(a)) correlate with larger population sizes of differentiated cells compared to higher values of *S* (Fig 11(b)). In the absence of hair follicles (taking *E* = 0, Fig 11(c-d)), we observe as before that the level of differentiated cells does not depend on *S*, but the variance decreases with the frequency of symmetric divisions. In Fig 12 we plot the relative standard deviation of the differentiated cells in the absence (*E* = 0) and in the presence (*E* > 0) of hair follicles, as functions of the symmetry of divisions. We can see that in the absence of hair follicles, the fluctuations are smallest for symmetric divisions (as discussed before, this is due to a decrease in *Var*(*J*)). On the contrary, in the presence of hair follicles, fluctuations are minimized by asymmetric divisions; the larger population sizes at small *S* contribute to the effect. We can speculate that the “No hair follicles” line simulates the evolutionary pressures in the footpad, resulting in an increase of *S*, and the “Hair follicle” line corresponds to the ear or tail epidermis, which favors more asymmetric divisions.

The two factors promoting symmetric divisions that are described above (the presence of micro-injuries and the lack of hair follicles in the footpad) can be viewed as two manifestations of the same mechanism. A general trend observed for control systems of type #5 can be expressed as follows: *in the presence of an exogenous source of SCs, asymmetric divisions correspond to the smallest relative size of fluctuations, and in the presence of random cell removal, symmetric divisions yield the smallest relative size of fluctuations.* In the text above we analyzed the two factors (micro-injuries and hair follicles) separately. It is not surprising that a model combining both factors gives the same predictions (see S1 Text).

## Discussion

In this paper we studied the role of symmetric and asymmetric divisions in the maintenance of tissue homeostasis. We have designed a general stochastic model that can be solved analytically to quantify how the amount of variation in the population size depends on various system parameters. We have shown that depending on the precise “wiring” of the controls in a control network, symmetric divisions can either stabilize or destabilize the system. In particular, among 5 minimal control loops identified in a two-compartment system [52], in two of them increasing the percentage of symmetric divisions will increase fluctuations, and in the remaining three it will decrease fluctuations, leading to an increased stability.

In the context of our study, mouse epidermis is an ideal model system for examining principles and mechanism of autonomous lineage control:

- Epidermal lineage is relatively simple and consists of basal SCs and suprabasal non-SC progenies;
- Epidermal proliferation can be easily studied due to skin accessibility, its two-dimensionality and with the help of multiple epidermis-specific genetic mouse models;
- Epidermal lineage strategy switches from all symmetric between embryonic days E8.5-E13.5, to mixed asymmetric + SC-generating symmetric in post- E13/5 mouse embryos, to fully mixed mode in adult mice;
- Signaling mechanism of epidermal cell fate selection has been partially elucidated, and appears to mainly rely on autonomous regulation by neighboring basal SCs and, possibly, non-SC suprabasal progenies.

Using our model, we studied the role of divisions symmetry in mouse epidermis. In particular, we focussed on the data on the percentage of symmetric divisions in different anatomic regions of the skin. While in the ear and tail epidermis, 20–22% of all divisions are symmetric, in the footpad epidermis this percentage increases to 40%. We showed that in the control system that best characterizes the epidermal lineage regulation, increasing the percentage of symmetric divisions enables the cell population to respond to environmental changes associated with micro-injuries. Conversely, decreasing the percentage of symmetric divisions allows to minimize the relative fluctuation size in cell populations in the presence of an exogenous source of SCs.

This is relevant for the specific case-study of mouse epidermis. On the one hand, the footpad is characterized by a higher level of micro-injuries compared to the ear and tail epidermis. Indeed, footpad skin is exposed to a variety of mechanical stresses, including friction from running, scratching, burrowing, fighting. All of these likely increase the probability of micro-injury to suprabasal epidermal compartment, and elevate the rate of cell loss from the lineage as compared to other, better protected anatomical areas, such as back skin, tail, ears. Our analysis suggests that the increased percentage of symmetric divisions in the footpad may be an adaptation to stabilize the tissue that faces the highest rate of micro-injuries from friction and abrasions. On the other hand, the footpad is characterized by the lack of hair follicles. The presence of hair follicles in the other regions such as ear and tail serves as an extra source of SCs. Our model shows that in the presence of such a source, asymmetric divisions are optimal from the point of view of keeping the size fluctuations low.

Our work adds to the discussion of the role of symmetry in the maintenance and dynamics of SC lineages. In [3], cellular strategies are considered in the context of homeostasis maintenance. It is stated that the balance between cell proliferation, differentiation and death can be achieved in two ways. On the one hand, it can be “obtained at the level of a single SC, which divides strictly into a new SC and a progenitor.” On the other hand, this “balance can also be achieved at the level of the SC population. Some SCs might be lost due to differentiation or damage, whereas others divide symmetrically to fill this gap.” The following question is raised in [3]: Why should mechanisms of tissue maintenance so often lean toward symmetric SC self-renewal? One possible answer comes from the ability of all symmetrically-dividing SCs to efficiently respond to injury and correct for lineage depletion. It however could be argued that the symmetric divisions are turned on in response to a sudden significant loss of cells, while the asymmetric division strategy can be employed in the course of normal homeostasis. In [100] we addressed a possible role of symmetric SC divisions as a cancer prevention mechanism. It was argued that symmetric divisions may slow down the accumulation of double-hit mutants, thus delaying the onset of many cancers, which depend on the inactivation of several tumor suppressor genes. In the present paper, we study cell division patterns from a different prospective, by looking at the maintenance of healthy tissues at homeostasis.

In general, each trend or strategy that has evolved in an organism, has been subject to a large number of selection pressures. In this paper we focus on only one type of selection pressure, namely, the pressure to keep the fluctuations down for an increased tissue stability (in [100] we focused on the selection pressure to delay the generation of cancerous mutations). In the case of footpad, both of these favor symmetric divisions, at least in the context considered in the two studies. There are however many other aspects of the evolutionary process that are not taken into account here.

One class of factors not included in the model is the true anatomical constraints of the epidermis. The basal compartment of the epidermis is limited in size and thus crowded, and the signaling mechanism regulating SC vs. differentiated cell decision-making is subject to physical constraints. Specifically, in real-life situations, the divisions of epidermal SCs can be truly symmetric if the mitotic axis is parallel to skin surface (i.e. both daughter cells remain in the basal layer). However, published data show that in adult epidermis, mitotic axis is randomly determined (horizontal, vertical and anywhere in-between) and commonly as the result of this one daughter cell is forced into the suprabasal layer, where it immediately experiences low WNT and high Dkk1 signaling that promotes its differentiation. Thus, these types of SC divisions are “forced” to be asymmetric. This mechanism alone will likely considerably limit the number of truly symmetric divisions. This can explain why 100% of symmetric divisions shown in our model is not realistic, since the model does not account for the real-life anatomic constrains of the skin.

The resulting solution found in the real-life epidermis is a trade-off between the anatomical and physical constraints, and possible evolutionary pressures, such as the ones described here. In the example worked out in this paper we show that symmetric divisions are more important for the footpad epidermis than they are for the ear and the tail. As an important future direction, a model with a more realistic 3D representation of cells in their niches, that describes the alignment and the geometry of SC divisions, could be created to combine the trends found here and anatomical considerations. A step in that direction has been made in [101], where a bi-compartmental SC niche was considered. In such a niche, one compartment is at the interface with the differentiated progeny and the other compartment is spatially separated from the differentiated cells. Further complexity can be added by explicitly modeling a spatially distributed system.

There are several other extensions of this work that are natural. While the mouse epidermis can be described as a two-compartmental lineage system, other tissues are characterized by a larger number of cell types of different degrees of differentiation (prominently, hematopoietic lineage). An extension of the current formalism to multi-compartment systems can be done by using the methodology developed in [102]. Further, the current model can only handle near-equilibrium situations. A different approach is required to study significant injuries and wound healing.

## Methods

### Stochastic model formulation

A stochastic model of cell population renewal is considered (see [52, 53]). The cells are subject to the following changes in a Poisson process with an infinitesimally small time-increment, Δ*t*:

- With probability *L*_*I*, *J*_ Δ*t* a SC divides. Divisions can be symmetric (with probability *S*_*I*, *J*_) or asymmetric (with probability 1 − *S*_*I*, *J*_).
  - With probability *L*_*I*, *J*_ *S*_*I*, *J*_ *P*_*I*, *J*_ Δ*t* a SC differentiation takes place resulting in a creation of two differentiated cells, (*I*, *J*)→(*I* − 1, *J* + 2).
  - With probability *L*_*I*, *J*_ *S*_*I*, *J*_(1 − *P*_*I*, *J*_)Δ*t* a SC proliferation takes place resulting in a creation of a SC, (*I*, *J*)→(*I* + 1, *J*).
  - With probability *L*_*I*, *J*_(1 − *S*_*I*, *J*_)Δ*t* a SC undergoes an asymmetric division resulting in a creation of a differentiated cell, (*I*, *J*)→(*I*, *J* + 1).
- With probability *D*_*I*, *J*_ Δ*t*, a differentiated cell dies, (*I*, *J*)→(*I*, *J* − 1).

A deterministic model that captures these events can be expressed as the following system of ordinary differential equations:

$$\begin{array}{ccc} \dot{x} & = & LS(1-P)-LSP=LS(1-2P), \end{array}$$ (13)

$$\begin{array}{ccc} \dot{y} & = & 2LSP+L(1-S)-D, \end{array}$$ (14)

where *x* and *y* refer to the numbers of stem and differentiated cells, and *L*, *P*, and *S* are all functions of *x* and *y*.

The stochastic description in terms of the Kolmogorov forward equation is given by the following equation for the variable *φ*_*I*, *J*_(*t*), the probability to find the system in state (*I*, *J*) at time *t*:

$$\begin{array}{ccc} {\dot{\varphi }}_{I,J} & = & \varphi_{I+1,J-2}L_{I+1,J-2}S_{I+1,J-2}P_{I+1,J-2}+\varphi_{I-1,J}L_{I-1,J}S_{I-1,J}\left(1-P_{I-1,J}\right)\\ & + & \varphi_{I,J-1}L_{I,J-1}\left(1-S_{I,J-1}\right)+\varphi_{I,J+1}D_{I,J+1}-\varphi_{I,J}\left(L_{I,J}+D_{I,J}\right), \end{array}$$ (15)

where the processes of the right hand side are presented in the same order as they appear in the list above. Note that system Eqs (13 and 14) is the “macroscopic law” obtained at the zeroth order of the “linear noise approximation” [52, 53].

We are interested in deriving equations for the mean values of the cell populations and their variances. To do this, we first define the steady states of the system, (*i*_0_, *j*_0_), by the following equations (which are obtained by solving Eqs (13) and (14)):

$$\begin{array}{c} L_{i_{0},j_{0}}=D_{i_{0},j_{0}}=L_{*},\;P_{i_{0},j_{0}}=\frac{1}{2},\;S_{*}=S_{i_{0},j_{0}}.\\ \left(\;mixed\;divisions\;steady\;state\right) \end{array}$$ (16)

$$\begin{array}{c} L_{i_{0},j_{0}}=D_{i_{0},j_{0}}=L_{*},\;S_{i_{0},j_{0}}=0,\;P_{*}=P_{i_{0},j_{0}}.\\ \left(\;purely\;asymmetric\;divisions\;steady\;state\right) \end{array}$$ (17)

Both equilibria are characterized by a balance between divisions and deaths (the first equation in Eqs (16) and (17)). In the first (mixed divisions) equilibrium, the probability of differentiation events is equal to the probability of proliferation events, thus ensuring that the expected change in the number of SCs is zero. The first two equations in Eq (16) define the equilibrium population sizes *i*_0_ and *j*_0_. The fraction of symmetric divisions, *S*_*I*, *J*_, does not influence the solution for *i*_0_ and *j*_0_, but, as shown below, can affect its stability properties and the size of fluctuations in the system.

The second (purely asymmetric) equilibrium is attained if the fraction of symmetric divisions can be made zero. The population sizes are determined by the first two equations in Eq (17), and the probability of differentiations, formally defined by the last equation, becomes irrelevant at equilibrium. Below we focus on the mixed divisions steady state. Calculations pertaining to steady state Eq (17) can be found in S1 Text.

### Stability analysis and variance calculations

The methodology presented here is based on the assumption of weak dependencies of the functions *L*_*I*, *J*_, *D*_*I*, *J*_, etc on their variables. It is developed in [52] and justified rigorously in [53]. Let us use the symbol *Z*_*I*, *J*_ to denote any of the functions *L*_*I*, *J*_, *P*_*I*, *J*_, *D*_*I*, *J*_, and *S*_*I*, *J*_. Suppose that we can represent the functions *Z*_*I*, *J*_ near the equilibrium as *Z*_*I*, *J*_ = *Z*(*ϵI*, *ϵJ*), where the parameter *ϵ* ≪ 1 defines the weakness of the dependence. It is convenient to denote *x* = *ϵI*, *y* = *ϵJ*, *i* = *I* − *i*_0_, *j* = *J* − *j*_0_, then we can expand the functions *Z*_*I*, *J*_ around the steady state in Taylor series:

$$\begin{array}{c} Z_{I,J}=Z_{i_{0},j_{0}}+z_{x}i+z_{y}j+\frac{1}{2}\left(z_{xx}i^{2}+z_{yy}j^{2}+2z_{xy}ij\right)+\cdots , \end{array}$$ (18)

where the subscripts *x* and *y* denote the partial derivative of the function with respect to its argument, evaluated at (*i*_0_, *j*_0_), and *z*_*x*_ = *Z*_*x*_ *ϵ*, *z*_*xx*_ = *Z*_*xx*_ *ϵ* ^2^, etc. In this description, while constants *Z*_*x*_ = *O*(1), *Z*_*xx*_ = *O*(1), etc are all of order one, all the first derivatives *z*_*x*_, *z*_*y*_ contain a factor *ϵ*, and all the second derivatives *z*_*xx*_, *z*_*xy*_, *z*_*yy*_ contain a factor *ϵ* ^2^.

Define ${\tilde{\varphi }}_{i,j}=\varphi_{i+i_{0},j+j_{0}}=\varphi_{I,J}$, and ${\tilde{Z}}_{i,j}=Z_{i+i_{0},j+j_{0}}=Z_{I,J}$, then Eq (15) can be reformulated as:

$$\begin{array}{ccc} {\dot{\tilde{\varphi }}}_{i,j} & = & {\tilde{\varphi }}_{i+1,j-2}{\tilde{L}}_{i+1,j-2}{\tilde{S}}_{i+1,j-2}{\tilde{P}}_{i+1,j-2}+{\tilde{\varphi }}_{i-1,j}{\tilde{L}}_{i-1,j}{\tilde{S}}_{i-1,j}\left(1-{\tilde{P}}_{i-1,j}\right)\\ & + & {\tilde{\varphi }}_{i,j-1}{\tilde{L}}_{i,j-1}\left(1-{\tilde{S}}_{i,j-1}\right)+{\tilde{\varphi }}_{i,j+1}{\tilde{D}}_{i,j+1}-{\tilde{\varphi }}_{i,j}\left({\tilde{L}}_{i,j}+{\tilde{D}}_{i,j}\right). \end{array}$$ (19)

Using expansion Eq (18) in Eq (19), we can derive the moment equations for this system. In what follows, we use the following notations for the moments:

$$\begin{array}{c} X_{\alpha \beta }=\sum_{i,j}i^{\alpha }j^{\beta }{\tilde{\varphi }}_{i,j}\left(t\right). \end{array}$$ (20)

Multiplying Eq (19) by *i* and by *j*, performing a summation in the two indices, and keeping only the highest order terms in *ϵ*, we obtain equations for the first moments in steady-state:

$$\begin{array}{ccc} 0 & = & -2L_{*}S_{*}\left(p_{y}X_{01}+p_{x}X_{10}\right), \end{array}$$ (21)

$$\begin{array}{ccc} 0 & = & \left(2L_{*}S_{*}p_{x}+q_{x}\right)X_{10}+\left(2L_{*}S_{*}p_{y}+q_{y}\right)X_{01}. \end{array}$$ (22)

For the second moments we have:

$$\begin{array}{ccc} 0 & = & \left(S_{*}l_{x}+L_{*}s_{x}\right)X_{10}+\left(S_{*}l_{y}+L_{*}s_{y}\right)X_{01}-4L_{*}S_{*}\left(p_{y}X_{11}+p_{x}X_{20}\right)+L_{*}S_{*},\\ 0 & = & -\left(S_{*}l_{x}+L_{*}s_{x}+2L_{*}S_{*}p_{x}\right)X_{10}-\left(S_{*}l_{y}+L_{*}s_{y}+2L_{*}S_{*}p_{y}\right)X_{01}\\ & + & 2L_{*}S_{*}\left[p_{x}X_{20}-p_{y}X_{02}+\left(p_{y}-p_{x}\right)X_{11}-1/2\right]+q_{y}X_{11}+q_{x}X_{20},\\ 0 & = & \left[S_{*}\left(4L_{*}p_{x}+l_{x}\right)+l_{x}+d_{x}+L_{*}s_{x}\right]X_{10}+\left[S_{*}\left(4L_{*}p_{y}+l_{y}\right)+l_{y}+d_{y}+L_{*}s_{y}\right]X_{01}\\ & + & \left(4L_{*}S_{*}p_{x}+2q_{x}\right)X_{11}+\left(4L_{*}S_{*}p_{y}+2q_{y}\right)X_{02}+L_{*}\left(2+S_{*}\right). \end{array}$$ (23)

Solving this system, we can obtain the expressions for the means and variances: $E\left[I\right]=X_{10}+i_{0}=i_{0},E\left[J\right]=X_{01}+j_{0}=j_{0};Var\left[I\right]=X_{20}-X_{10}^{2}$, $Var\left[J\right]=X_{02}-X_{01}^{2}$. The highest order terms for the variances are given by

$$\begin{array}{c} Var\left[I\right]=\frac{K_{x}}{4B\Delta },\;Var\left[J\right]=\frac{K_{y}}{4B\Delta }, \end{array}$$ (24)

where we defined the quantities:

$$\begin{array}{ccc} \Delta & = & q_{x}p_{y}-q_{y}p_{x}, \end{array}$$ (25)

$$\begin{array}{ccc} B & = & 2L_{*}S_{*}\left(p_{x}-p_{y}\right)-q_{y}, \end{array}$$ (26)

$$\begin{array}{ccc} K_{x} & = & 2L_{*}S_{*}\Delta +q_{y}^{2}+8L_{*}^{2}S_{*}p_{y}^{2}, \end{array}$$ (27)

$$\begin{array}{ccc} K_{y} & = & 2L_{*}\left(2+S_{*}\right)\Delta +q_{x}^{2}+8L_{*}^{2}S_{*}p_{x}^{2}. \end{array}$$ (28)

Details of stability analysis are given in S1 Text. It follows that mixed division steady state is stable as long as Δ > 0 and *B* > 0; constants *K*_*x*_ and *K*_*y*_ are always positive quantities. Increasing Δ and *B* makes the system more robust by decreasing the variation of population sizes. A *Mathematica* file is provided in S1 File that calculates result Eq (24) symbolically.

#### Notes

There are two important conclusions from the above analysis.

- The numbers of stem and differentiated cells at the equilibrium do not depend on the quantity *S* (the fraction of symmetric divisions). This is because regardless of the proportion of symmetric divisions, there are only two requirements for the constancy of the population: (1) Probability of differentiation under symmetric divisions is 1/2 (this keeps the number of stem cells constant), and (2) The rate of divisions equals to the rate of death (this keeps the number of differentiated cells constant). Both conditions are independent of *S*.
- Related to this, the fraction of symmetric divisions, *S*, only enters the expressions for the cell number variances. In the first order analysis above, only the equilibrium value, S_*_, and not the derivatives, appear in the expressions for the second moments.

Below we explore how the probability of symmetric divisions affects the homeostatic control.

### The role of asymmetric divisions in cell number regulation

The equilibrium values for the numbers of stem and differentiated cells are unaffected by the presence of asymmetric divisions, as illustrated by Eq (16). On the other hand, the probability of symmetric divisions, S_*_, can influence two important properties of the SC system: (a) stability of the equilibrium and (b) the size of fluctuations (the amount of variance), which is related to the robustness of homeostatic control.

#### Stability

The only way in which the fraction of symmetric divisions can influence stability of the system is by changing the sign of the quantity *B*, Eq (26). From Eq (26):

$$\begin{array}{c} B>0\Leftrightarrow S_{*}>S_{c}\equiv \frac{q_{y}}{2L_{*}\left(p_{x}-p_{y}\right)} \end{array}$$ (29)

If the value *S*_*c*_ is between 0 and 1, then we have the following trends:

- Increasing the fraction of asymmetric divisions can destabilize the system if *q*_*y*_ < 2L_*_(*p*_*x*_ − *p*_*y*_) and *p*_*x*_ > *p*_*y*_;
- Increasing the fraction of symmetric divisions can destabilize the system if *q*_*y*_ < 0 and *p*_*x*_ < *p*_*y*_.

#### The size of fluctuations (robustness)

In order to study the influence of asymmetric divisions on the behavior of cell populations, we consider the derivatives of the variances of *I* and *J* with respect to parameter S_*_:

$$\begin{array}{ccc} \frac{d\;Var[I]}{dS_{*}} & = & \frac{L_{*}}{2B^{2}\Delta }p_{y}q_{y}\left(q_{y}-q_{x}-4L_{*}p_{y}\right), \end{array}$$ (30)

$$\begin{array}{ccc} \frac{d\;Var[J]}{dS_{*}} & = & \frac{L_{*}}{2B^{2}\Delta }\left(p_{x}q_{x}-\Delta \right)\left(q_{y}-q_{x}-4L_{*}p_{y}\right). \end{array}$$ (31)

The signs of these derivatives can be different, depending on parameters. For a fixed set of parameters, the dependence on S_*_ is monotonic, that is, each of the variances either grows or decays with S_*_.

#### Application to the five minimal controls

- Two-control model #1, Fig 3. In this case, *S*_*c*_ = 0 from Eq (29), therefore the steady state is stable for any S_*_ > 0. Further, we have
  $$\begin{array}{c} \frac{d\;Var[I]}{dS_{*}}=0,\;\frac{d\;Var[J]}{dS_{*}}=\frac{L_{*}}{2B^{2}}\left(q_{x}+4L_{*}p_{y}\right)<0. \end{array}$$ (32)
  In other words, increasing the share of symmetric divisions reduces the fluctuation size in the system. Thus, symmetric divisions (i.e. S_*_ = 1) will be optimal for this system.
- Two-control model #2, Fig 3. In this case, *S*_*c*_ < 0 from Eq (29), therefore the steady state is stable for any value of S_*_. Further, we have
  $$\begin{array}{c} \frac{d\;Var[I]}{dS_{*}}=0,\;\frac{d\;Var[J]}{dS_{*}}=\frac{L_{*}}{2B^{2}}\left(-q_{y}\right)>0. \end{array}$$ (33)
  That is, increasing the share of asymmetric divisions makes the fluctuations smaller. Thus, purely asymmetric divisions (i.e. S_*_ → 0) will be optimal.
- Three-control model #3, Fig 3. In this case, we have
  $$\begin{array}{c} \frac{d\;Var[I]}{dS_{*}}>0,\;\frac{d\;Var[J]}{dS_{*}}>0. \end{array}$$ (34)
  For the first three-control minimal system, *p*_*x*_ < *p*_*y*_ and *q*_*y*_ < 0. From the viewpoint of stability, it is disadvantageous to increase the value of S_*_. From the perspective of robustness, small values of S_*_ are best, since *Var*[*I*] and *Var*[*J*] grow with it. Thus, asymmetric divisions will be optimal.
- Three-control model #4, Fig 3. In this case, we have *p*_*x*_ > *p*_*y*_ and *q*_*y*_ < 2L_*_(*p*_*x*_ − *p*_*y*_). Therefore, decreasing the value of S_*_ may destabilize the system. Further, we have
  $$\begin{array}{c} \frac{d\;Var[I]}{dS_{*}}<0,\;\frac{d\;Var[J]}{dS_{*}}<0, \end{array}$$ (35)
  that is, fluctuations decay with S_*_. Thus, symmetric divisions will be optimal.
- Three-control model #5, Fig 3. Again, *p*_*x*_ > *p*_*y*_ and *q*_*y*_ < 2L_*_(*p*_*x*_ − *p*_*y*_), and
  $$\begin{array}{c} \frac{d\;Var[I]}{dS_{*}}=0,\;\frac{d\;Var[J]}{dS_{*}}<0. \end{array}$$ (36)
  Therefore, as in the previous case, symmetric divisions will be optimal.

## Supporting Information

S1 Text Technical details of the mathematical analysis. Both analytical calculations and numerical simulations are explained.
(PDF)

S1 File A *Mathematica* file that calculates the variances of cell populations symbolically. Quantities *Var*[*I*] and *Var*[*J*] are calculated both for the mixed divisions equilibrium and for the purely asymmetric equilibrium.
(NB) Click here for additional data file.
