# Supplementary material for: The Role of Symmetric Stem Cell Divisions in Tissue Homeostasis
Source: PLoS Comput Biol. 2015 Dec 23;11(12):e1004629. doi: 10.1371/journal.pcbi.1004629 (PMC4689538; doi:10.1371/journal.pcbi.1004629)
Supplement: S1 Text — Both analytical calculations and numerical simulations are explained. (PDF) [file pcbi.1004629.s001.pdf]

Text S1

The role of asymmetric stem cell divisions in tissue homeostasis

Jienian Yang, Maksim V. Plikus, and Natalia L. Komarova

Contents

1 Minimal control systems **1**

2 Stability analysis **2**

3 Purely asymmetric divisions steady state **3**
3.1 Results for the cell number means and variances 3
3.2 A case study with a purely symmetric divisions equilibrium . . 4

4 Numerical simulations **7**

5 Modeling micro-injuries **8**
5.1 Loss of differentiated cells 8
5.2 Loss of SCs 10
5.3 Numerical examples 11

6 Modeling the effect of hair follicles **15**
6.1 An exogenous source of SCs 15
6.2 Combining the effects of the hair follicles and micro-injuries . 17

1 Minimal control systems

In the section we review the analysis of Komarova (2013) in the context of the present work, see also figure 3 of the main text. For the two-compartment model, we note that at least two of the four quantities, (q_x, q_y, p_x, p_y), must be nonzero to satisfy the stability condition: $\Delta \equiv p_y q_x - p_x q_y > 0$. In fact, there are exactly two cases where only two of the four derivatives are nonzero and satisfy the other stability condition: $B \equiv 2L_* S_*(p_x - p_y) - q_y > 0$:

#1 $q_x < 0, \quad p_y < 0, \quad q_y = p_x = 0;$

#2 $q_y < 0, \quad p_x > 0, \quad q_x = p_y = 0.$

Extending the analysis to three nonzero controls, we find that there are exactly three cases that satisfy both stability conditions:

#3 $q_y < 0, \quad q_x > 0, \quad 0 < p_y < -\frac{q_y}{2L_*S_*}, \quad p_x = 0;$

#4 $q_y > 0, \quad q_x = 0, \quad p_x < 0, \quad p_y < p_x - \frac{q_y}{2L_*S_*} < 0;$

#5 $q_y = 0, \quad q_x > 0, \quad p_x > p_y > 0.$

Note that the case where $p_y = 0$ yields a system of controls that is reducible to the two-control model #2 by setting $q_x = 0$.

2 Stability analysis

A deterministic description of the system is given by equations

$$\dot{x} = LS(1-P) - LSP = LS(1-2P), \tag{1}$$
$$\dot{y} = 2LSP + L(1-S) - D, \tag{2}$$

The equilibria are defined by

$$L_{i_0,j_0} = D_{i_0,j_0} = L_*, \quad P_{i_0,j_0} = \frac{1}{2}, \quad S_* = S_{i_0,j_0}. \tag{3}$$
$$L_{i_0,j_0} = D_{i_0,j_0} = L_*, \quad S_{i_0,j_0} = 0, \quad P_* = P_{i_0,j_0}. \tag{4}$$

We can compute the Jacobian of the system, evaluated at mixed divisions steady state (3):

$$J = \begin{pmatrix} \frac{\partial f}{\partial x} & \frac{\partial f}{\partial y} \\ \frac{\partial g}{\partial x} & \frac{\partial g}{\partial y} \end{pmatrix},$$

where $f = LS(1-2P), g = 2LSP + L(1-S) - D$, and all derivatives are evaluated at the equilibrium (i_0, j_0).

Let det, τ be the determinant and the trace of J, respectively. Then,

$$\begin{aligned} det &= \frac{\partial f}{\partial x} \cdot \frac{\partial g}{\partial y} - \frac{\partial f}{\partial y} \cdot \frac{\partial g}{\partial x} \\ &= \frac{2L_*S_*(q_x p_y - q_y p_x)}{\epsilon^2}; \\ \tau &= \frac{\partial f}{\partial x} + \frac{\partial g}{\partial y} = -\frac{2L_*S_*(p_x - p_y) - q_y}{\epsilon}. \end{aligned}$$

The stability of the system requires $det > 0$ and $\tau < 0$. It follows that mixed divisions steady state is stable as long as $\Delta > 0$ and $B > 0$, where we defined

$$\Delta = q_x p_y - q_y p_x, \quad B = 2L_* S_* (p_x - p_y) - q_y.$$

Similarly, we evaluate the Jacobian at purely asymmetric divisions steady state (4), and we obtained in this case:

$$\begin{aligned} det &= \frac{L_*(2P_* - 1)(q_x s_y - q_y s_x)}{\epsilon^2}; \\ \tau &= \frac{L_*(s_y - s_x)(2P_* - 1) + q_y}{\epsilon}. \end{aligned}$$

It follows that purely asymmetric divisions steady state is stable as long as $\delta > 0$ and $b > 0$, where

$$b = -L_*(s_y - s_x)(2P_* - 1) - q_y, \quad \delta = (2P_* - 1)(q_x s_y - q_y s_x).$$

3 Purely asymmetric divisions steady state

The analysis of the mixed divisions steady state is presented in the main text, and the result for the variances is given by

$$Var[I] = \frac{K_x}{4B\Delta}, \quad Var[J] = \frac{K_y}{4B\Delta}. \tag{5}$$

Here we present analysis of the dynamics in the vicinity of the purely asymmetric divisions equilibrium, equation (4).

3.1 Results for the cell number means and variances

It is important to note that in the limit of $S_* \to 0$ (asymmetric divisions only) formulas (5) break down. When $S_* = 0$, the numbers of stem cell cannot change in the model. The state space becomes one-dimensional. By using the same approach of "linear noise approximation" (Yang *et al.*, 2015), we can obtain the means and the variances of the cell population to the highest order:

$$\begin{aligned} E[I] &= i_0, \quad E[J] = j_0; & (6) \\ Var[I] &= \frac{\{L_*(2P_* - 1)s_y\}^2}{b\delta}, & (7) \\ Var[J] &= \frac{L_*}{b} + \frac{\{L_*(2P_* - 1)s_x\}^2}{b\delta}. & (8) \end{aligned}$$

We provide a *Mathematica* file to compute the means and the variances given above. Evaluating this at the equilibrium, we obtain the correct answer for strictly asymmetric divisions (by taking the limit $S_* \to 0$):

$$Var[I] = 0, \quad Var[J] = -\frac{L_*}{q_y}. \tag{9}$$

3.2 A case study with a purely symmetric divisions equilibrium

In this section, we will demonstrate that the analytic results of section 3.1 agree with the numerical results. We will use the example of two-control model #2 in figure 3 of the main text, and equip it with a control of division symmetry parameter, S. This model is characterized by negative control on division and positive control on differentiation. We consider the following functional forms:

$$\begin{aligned} L(x,y) &= \frac{1}{1+y}, \quad P(x,y) = 0.7 \cdot \tanh(x), \\ D(x,y) &= 1 - L(x,y), \quad S(x,y) = \Big(1 - \frac{1}{cx}\Big)^2, \end{aligned} \tag{10}$$

where c is a positive constant.

The equilibria. We have $q_x = p_y = s_y = 0$, $q_y = -2\epsilon(1+y)^{-2} < 0$, $p_x = 0.7\epsilon \cdot \mathrm{sech}^2(x) > 0$, and $s_x = 2\epsilon\Big(1 - \frac{1}{cx}\Big) \cdot \frac{1}{cx^2}$. The mixed divisions steady state can be obtained by solving $P(x,y) = 1/2$, and $L(x,y) = D(x,y)$:

$$x_m = \frac{\log(6)}{2}, \quad y_m = 1 \ (or\ i_m = \frac{\log(6)}{2\epsilon}, \quad j_m = \frac{1}{\epsilon}). \tag{11}$$

The purely asymmetric divisions steady state can be obtained by solving $S(x,y) = 0$, and $L(x,y) = D(x,y)$:

$$x_a = \frac{1}{c}, \quad y_a = 1 \ (or\ i_a = \frac{1}{c\epsilon}, \quad j_a = \frac{1}{\epsilon}). \tag{12}$$

By using the theory in the main text, we can obtain the means and the

variances for the mixed divisions solution:

$$E[I] = \frac{\log(6)}{2\epsilon}, \quad (13)$$

$$E[J] = \frac{1}{\epsilon}, \quad (14)$$

$$Var[I] = \frac{2L_*S_*\Delta + q_y^2}{4B\Delta}, \quad (15)$$

$$Var[J] = \frac{2L_*(2+S_*)\Delta + 8L_*^2S_*p_x^2}{4B\Delta}, \quad (16)$$

where all the partial derivates are evaluated at the mixed divisions steady state, and $L_* = 1/2$, $S_* = (1 - \frac{2}{c\log(6)})^2$, $\Delta = -q_y p_x$, and $B = 2L_*S_*p_x - q_y$.

Similarly, we obtain the means and the variances for the purely asymmetric solution by equations (6-8):

$$E[I] = \frac{1}{c\epsilon}, \quad E[J] = \frac{1}{\epsilon}, \quad (17)$$

$$Var[I] = 0, \quad Var[J] = -\frac{L_*}{q_y}. \quad (18)$$

where $L_* = 1/2$, and q_y is evaluated at the purely asymmetric equilibrium.

Stability analysis. The mixed solution is stable in this case (since $det > 0$, and $\tau < 0$), but the stability of asymmetric solution is ambiguous (since $det = 0$, and $\tau < 0$), see Section 2. Nevertheless, a nonlinear stability analysis can be performed in this case. Let $c_* = 1/x_m$, then if $c > c_*$, $x_m > x_a$, and if $c < c_*$, $x_m < x_a$. We have the following results by nonlinear stability analysis:

1. If $c > \frac{2}{\log(6)}$, and the system starts near (x_a, y_a) with $x > \frac{1}{c}$, then the solution will converge to (x_m, y_m) from below.
2. If $c > \frac{2}{\log(6)}$, and the system starts near (x_a, y_a) with $x < \frac{1}{c}$, then the solution will converge to (x_a, y_a) from below.
3. If $c < \frac{2}{\log(6)}$, and the system starts near (x_a, y_a) with $x > \frac{1}{c}$, then the solution will converge to (x_a, y_a) from above.
4. If $c < \frac{2}{\log(6)}$, and the system starts near (x_a, y_a) with $x < \frac{1}{c}$, then the solution will converge to (x_m, y_m) from above.

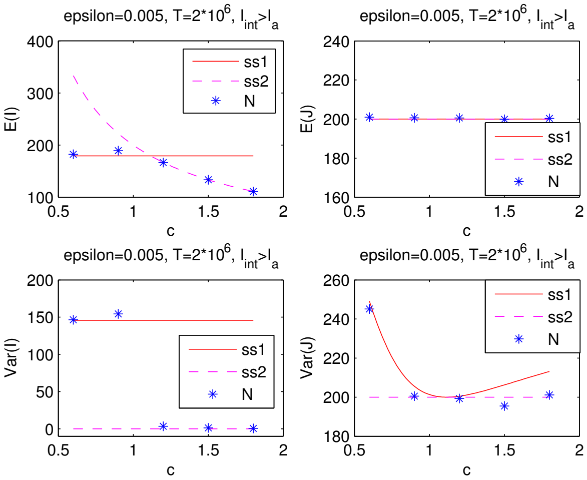

Figure S1: The behavior of the means and variances of the system described by (10) with $\epsilon = 0.005$. The system starts at $(\frac{3i_m+i_a}{4}, j_a)$ for $c < c_* = 2/\log(6)$, and it starts at $(i_a - 20, j_a)$ when $c > c_*$. The analytical results given by (13-18) (solid line for mixed division and dashed line for purely asymmetric division) are compared with numerical results (stars), for different values of c. ('ss1') stands for the mixed division solution, ('ss2') stands for the purely asymmetric division solution, and ('N') stands for the numerical results.

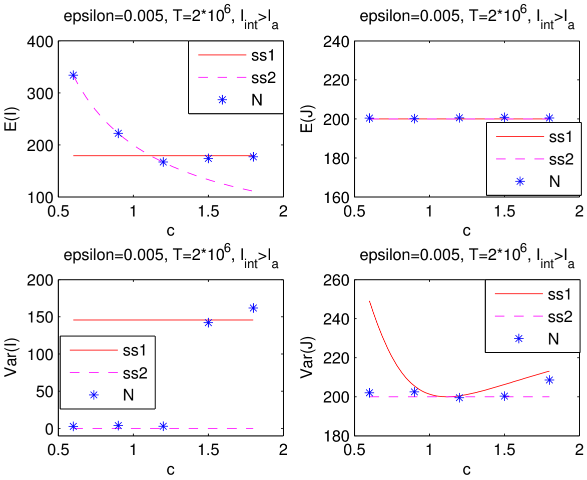

Figure S2: Analogous graph as figure S1, except the system starts at $(i_a + 10, j_a)$ for $c < c_*$, and it starts at $(\frac{3i_m+i_a}{4}, j_a)$ for $c > c_*$.

Numerical results. For each set of ϵ and c, we ran numerical simulations starting near (x_a, y_a), and finishing either when the number of time steps reached $2 \cdot 10^6$, or if any of the cell types went extinct. We then computed the means and the variances of the cell population over the time-course of each simulation. From figure S1, we observe that when the system starts near (x_a, y_a) with $x < \frac{1}{c}$, the numerical results agree with the analytic results given by cases (2) and (4) in the above stability analysis. As observed in figure S2, the numerical results are consistent with the analytic results given by cases (1) and (3) in the stability analysis when the system starts near (x_a, y_a) with $x > \frac{1}{c}$.

4 Numerical simulations

The numerical simulations presented in this paper are set up in the following way. At each time-step, one of two events happen: either a SC divides with probability $\frac{L(i,j)}{L(i,j)+D(i,j)}$, or a differentiated cell dies with probability $\frac{D(i,j)}{L(i,j)+D(i,j)}$. In the case of a SC division, its nature is determined based on the probabilities $S(i,j)$ and $P(i,j)$. In this algorithm, each time-step corresponds to a cellular event (a division or a death). In other words, we have a non-uniform clock which only advances if a biological event takes place.

This is in contrast with a real-time, physical clock, which continues "ticking" even if no events take place. If we were to implement the latter, more realistic simulation, we could use the well-known Gillespie algorithm, where the probabilities of different events are determined as above, but the time-steps between the events, Δt_i, are assigned according to an exponential distribution. The latter type of a simulation would present a picture of population dynamics as it happens in physical time.

For our purposes, however keeping track of biological time is unnecessary. We are only concerned with calculating the means and the variances of the cell population, which are the same in our simulation and in the real-time simulation just described. To show this, let us suppose that we need to calculate the mean value of a stochastic variable f_i, which stands for the population size of interest at time-step i of our simulation. Then the mean value obtained from our simulation after n time-steps is simply given by

$$\langle f \rangle_1 = \frac{\sum_{i=1}^{n} f_i}{n}.$$

The value obtained from the simulation which keeps track of biological time is given by

$$\langle f\rangle_2 = \frac{\sum_{i=1}^n f_i \Delta t_i}{\sum_{i=1}^n \Delta t_i}.$$

The two expressions are equal because

$$\frac{\sum_{i=1}^n f_i}{n}\frac{\sum_{i=1}^n \Delta t_i}{n} = \frac{\sum_{i=1}^n f_i \Delta t_i}{n},$$

where the left hand side is $\langle f\rangle_1\langle\Delta t\rangle_1$, and the right hand side is $\langle f\Delta t\rangle_1$. Because the two variables are independent, this equality holds.

The same argument holds for the moments of the variable f_i, where for kth moment we simply consider the mean value of the quantity $(f_i)^k$.

5 Modeling micro-injuries

Let us suppose that as a result of micro-injuries, both SCs and differentiated cells experience a certain level of death, which in the ODE framework can be expressed as the following modification of system (1-2):

$$\dot{x} = LS(1-P) - LSP - A\eta = LS(1-2P) - A\eta, \quad (19)$$

$$\dot{y} = 2LSP + L(1-S) - D - B\xi, \quad (20)$$

where functions $A\eta$ and $B\xi$ describe the effect of the micro-injuries on the populations of SCs and differentiated cells respectively, and in general can depend on x and y. It is convenient to use the constants A and B to measure the magnitude of the cell death.

5.1 Loss of differentiated cells

Let us start our general analysis with the case where $A = 0$. For example, removing a fixed fraction of the differentiated cell population (as modeled in figure 10 of the main text) corresponds to

$$A = 0, \quad \xi = y.$$

As long as $A = 0$, at steady state we have, as before, $P = 1/2$, and the equilibrium is defined by

$$P_{i_0,j_0} = 1/2, \quad L_{i_0,j_0} - D_{i_0,j_0} = \xi_{i_0,j_0}.$$

(compared with solution (3)). This shows that when SCs are not killed by micro-injuries, the percentage of symmetric divisions, S, does not influence the equilibrium. Let us denote the derivatives of the equilibrium values of SCs and differentiated cells with respect to S by x_S and y_S respectively. We have

$$x_S = y_S = 0.$$

The actual equilibrium values can be modified by the presence of the micro-injuries. To find out the dependence of the equilibrium number of cells on the intensity of cell death, we write down the equations that define the equilibrium of system (19-20):

$$P(x,y) = \frac{1}{2}, \quad (21)$$

$$L(x,y) - D(x,y) = B\xi(x,y). \quad (22)$$

To be specific, we assume the following dependencies of the functions of their variables at the equilibrium:

$$p_x > 0 \quad p_y > 0, \quad (23)$$

$$q_x > 0, \quad q_y = 0, \quad (24)$$

$$\xi_x = 0, \quad \xi_y > 0. \quad (25)$$

The first two lines above correspond to control system of type #5, and the last line assumes that the rate of differentiated cell removal growth with their numbers. Let us differentiate system (21-22) with respect to B, and denote the derivatives of the equilibrium numbers of SCs and differentiated cells with respect to B as x_B and y_B respectively. We have from the first equation

$$p_x x_B + p_y y_B = 0,$$

which means that x_B and y_B must have different signs. From equation (22) we obtain

$$y_B = \frac{-\xi}{B\xi_y + q_x p_y / p_x} < 0.$$

We conclude that the equilibrium number of SCs increases with the magnitude of the differentiated cell death rate, and the number of differentiated cells decreases. This trend was observed for example in the system studied in figure 10 of the main text. As the micro-injury intensity changed from 1% to 10% to 20%, the mean value of the differentiated cell population decreased. This was factored in the calculations of the relative standard deviation, figure 10(d) of the main text.

5.2 Loss of SCs

If SCs are directly affected (i.e. killed) by the micro-injury process, the situation changes. In this case, the population level at the equilibrium becomes a function of S. To demonstrate this, we write down the equations that define the equilibrium of system (19-20):

$$P(x,y) = \frac{1}{2} - \frac{1}{S}\frac{A\eta}{L}, \tag{26}$$

$$L(x,y) - D(x,y) = A\eta(x,y). \tag{27}$$

We will assume that conditions (23-24) hold, and add the following assumption:

$$\eta_x > 0, \quad \eta_y = 0,$$

that is, the rate of SC removal growth with their numbers. Differentiating equation (27) with respect to S, we obtain

$$q_x x_S = B\eta_x x_S,$$

which in the general case implies that

$$x_S = 0,$$

that is, the equilibrium number of SCs does not depend on the frequency of symmetric divisions. Further, differentiating (26) with respect to S, we obtain that

$$y_S = \frac{A\eta}{2LS^2 p_y} > 0. \tag{28}$$

In other words, the equilibrium number of differentiated cells increases with S. To investigate the dependence of the equilibrium values of the cell numbers on the level of SC death, A, one would need to differentiating equations (26-27) with respect to A, similar to the method used before.

The calculations presented here are illustrated by the following example. In figure S3 we assumed that in 0.1% of the updates, the number of SCs is reduced by 1%, that is, in system (19-20),

$$\eta = x, \quad B = 0,$$

where B is a constant. In figure S3(a) we set $c = 0$, which corresponds to purely asymmetric divisions. As can be seen, this system cannot tolerate

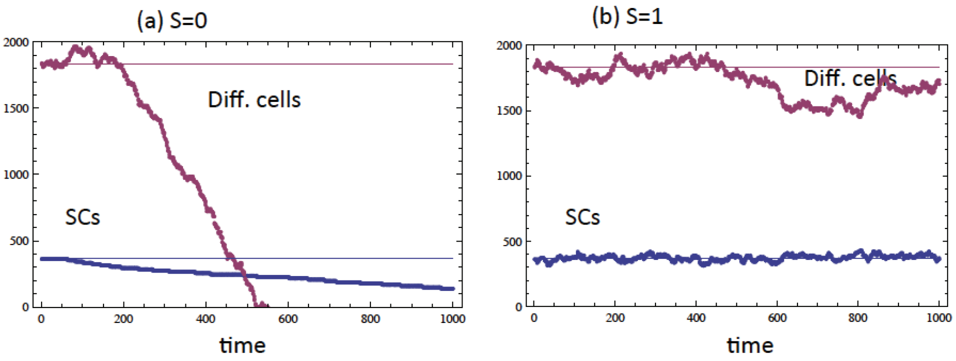

Figure S3: Modeling micro-injuries by introducing a 1% increase in the number of SCs in 0.1% of randomly chosen temporal updates. The numbers of differentiated and SCs are plotted as functions of time, for a typical run, with (a) $S = 0$ and (b) $S = 1$. Parameter $h = 0.3$ was used in equations (11-12) of the main text.

SCs death because there is no mechanism to replenish SCs. In this case, the number of SCs steadily decreases, and the population of differentiated cells also plunges. There is no biologically relevant steady state in this system. Figure S3(b) shows a very different behavior. Now, a significant percentage of SC divisions are symmetric. In such cases, the micro-injuries experienced by the system do not lead the a catastrophic decrease in cell numbers. Instead, the system finds a quasi-steady state solution. The levels of differentiated cells at equilibrium is an increasing function of S, see figure S4.

5.3 Numerical examples

Apart from the example presented in the main text (see figure 10 of the main text), we have tested several alternative models of micro- injuries. In one model, we start with the following specific control functions:

$$L_{I,J} = \frac{\tanh(\epsilon I)}{2\tanh(\epsilon I) + 0.4}, \quad P_{I,J} = \tanh(\epsilon I + 0.1\epsilon J), \tag{29}$$

$$D_{I,J} = h + 0.01\epsilon J, \quad S_{I,J} = c. \tag{30}$$

To model micro-injuries, instead of removing cells from the system, we increase the death rate of the differentiated cells in a fixed fraction of updates. Figure S6 demonstrates the resulting behavior. In figure S6(a), all divisions are asymmetric, and in figure S6(b) divisions are purely symmetric. Again, we can see that symmetric divisions lead to a smaller variance.

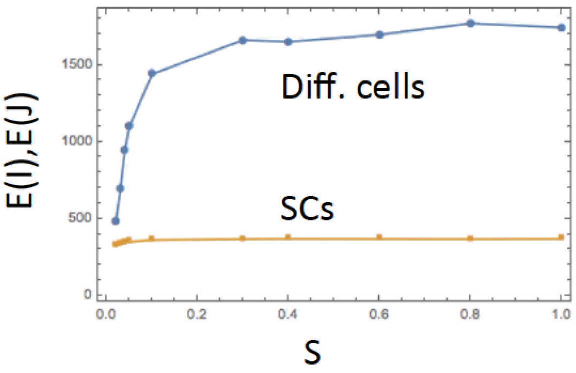

Figure S4: Modeling micro-injuries by introducing a 1% increase in the number of SCs in 0.1% of randomly chosen temporal updates. The expected population sizes of SCs and differentiated cells are plotted as functions of S, the frequency of symmetric divisions. Parameters are as in figure S3.

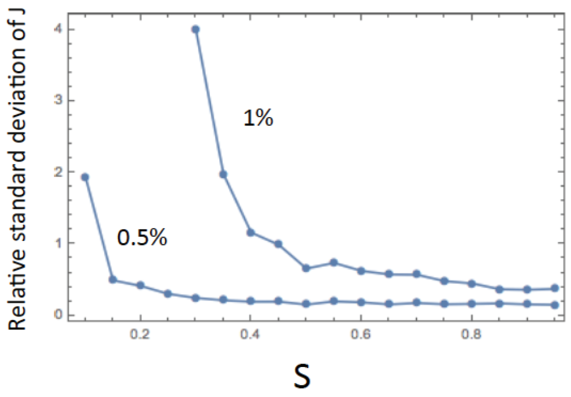

Figure S5: Modeling micro-injuries by introducing a small percent (1% or 0.5%) decrease in both SCs and differentiated cells in 0.5% or updates (randomly chosen). The relative standard deviation of the number of differentiated cells is plotted as a function of the proportion of symmetric divisions, S. The percentage decrease is marked above the lines. The rest of the parameters are as in figure S3.

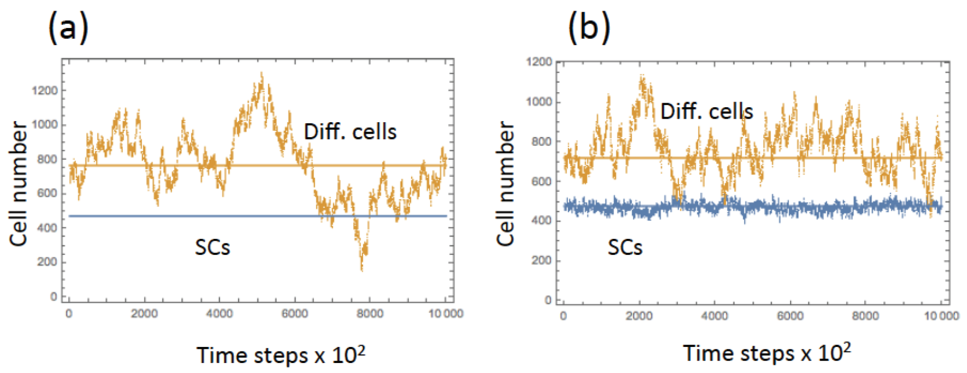

Figure S6: Modeling micro-injuries by increasing the death rate of differentiated cells in 40% of temporal updates (randomly chosen): 60% of the time, $h = 0.3$ is used, and 40% of the time, $h = 0.4$ is used in equation (30). The numbers of differentiated cells and SCs are plotted as functions of time, for a typical run. The percentage of symmetric divisions is 0 in (a) and 100% in (b). Increasing S from 0 to 1 for these parameters results in an approximately 3-fold decrease in the variance of the differentiated cell population.

In other variants of the model we also included a reduction in the number of SCs. We considered a model which included a small percentage decrease in both the numbers of SCs and differentiated cells, such that

$$\eta = x, \quad \xi = y, \quad A = B.$$

Again, in this case the steady state values of cells increase with S, as shown in equation 28. In figure S5 we show the relative standard deviation of the numbers of differentiated cells as a function of S, for two different levels of micro-injuries. As before, the relative size of fluctuations in the population is the largest for small values of S (asymmetric divisions).

Finally, we performed a study of a similar model with an additional random increase in the death rate of differentiated cells also included. Figure S7 shows simulations where at a fraction of temporal updates, we remove a small fraction of both SCs and differentiated cells, and also increase the death rate of SCs. In this case, increasing the symmetry of divisions does not only stabilize the system but also increases the steady state level of differentiated cells, as in equation 28.

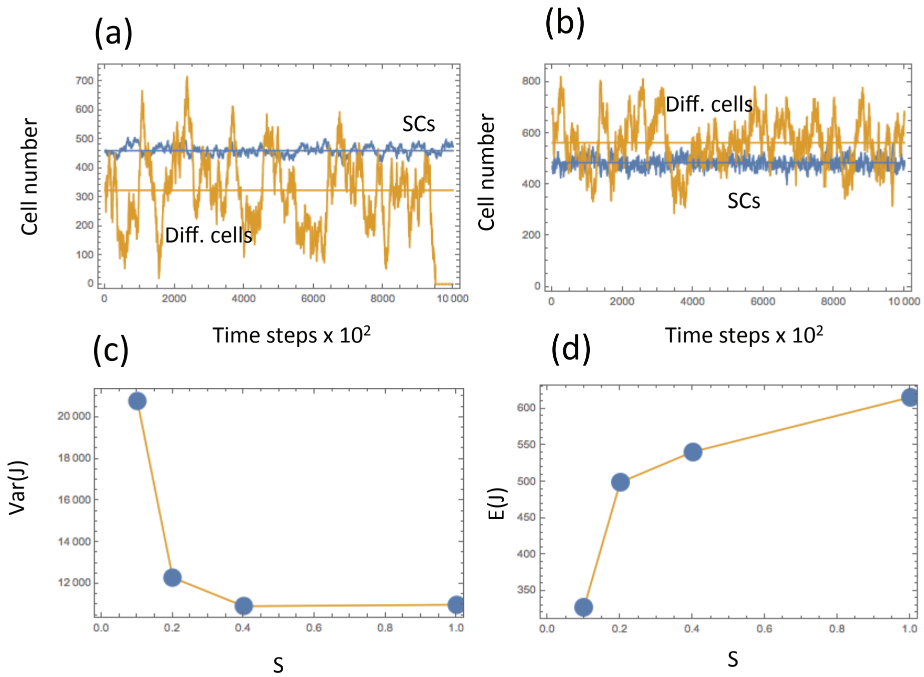

Figure S7: Modeling micro-injuries by introducing a 0.005% decrease in the number of both stem and differentiated cells in 20% of temporal updates (randomly chosen), and also increasing the death rate of differentiated cells from 0.3 to 0.5, equation (30). In (a,b) the numbers of SCs and differentiated cells is plotted as functions of time, for a typical run. In (a), most divisions are asymmetric ($S = 0.1$), and in (b), all divisions are symmetric ($S = 1$). (c,d) The variance and the mean of the number of differentiated cells as a function of the percentage of symmetric divisions.

6 Modeling the effect of hair follicles

6.1 An exogenous source of SCs

It can be argued that hair follicles can serve as alternative (backup) source of differentiated cells in haired skin, such as back skin, ear skin, and tail skin. Lack of hair follicles as a backup SC source puts all the "pressure" on epidermal SCs to compensate for all types of cell loss. To include the hair follicles as an exogenous source of SCs, we consider the following model:

$$\dot{x} = LS(1-P) - LSP + E - A\eta = LS(1-2P) + E - A\eta, \quad (31)$$
$$\dot{y} = 2LSP + L(1-S) - D - B\xi, \quad (32)$$

where $E(x,y)$ is the influx of SCs from the hair follicles. We will assume that

$$E_x < 0, \quad E_y = 0,$$

that is, the hair follicles respond to the lack of SCs in the epidermis by providing a back-up supply. In the simplest case where $A = B = 0$, we can use the previous analysis of system (26-27) to obtain that

$$x_S = 0, \quad y_S < 0.$$

In other words, the number of differentiated cells at the equilibrium decays with the frequency of symmetric divisions. This result holds for $A > 0$, as long as $E > A\eta$ at the equilibrium, that is, the input from the hair follicles is greater than the loss from SC death. (In the opposite case, where $E < A\eta$, the equilibrium number of the differentiated cells is a growing function of S.)

This may explain the tendency of ear/tail epidermis to have fewer symmetric divisions, compared to the footpad epidermis. In the tail/ear epidermis, hair follicles serve as an exogenous source of SCs, which corresponds to $E > A\eta$. In this case, the number of differentiated cells is highest for asymmetric divisions. On the contrary, in the footpad, hair follicles are absent, and in the presence of SC death, we have the opposite scenario where $E < A\eta$. Thus, the number of differentiated cells is the highest for $S = 1$, that is, the lack of hair follicles promotes symmetric SC divisions.

This argument works if we assume that (1) maximizing the equilibrium number of differentiated cells is a valid evolutionary objective, and (2) a certain level of SC death is present in the footpad. In the absence of SC

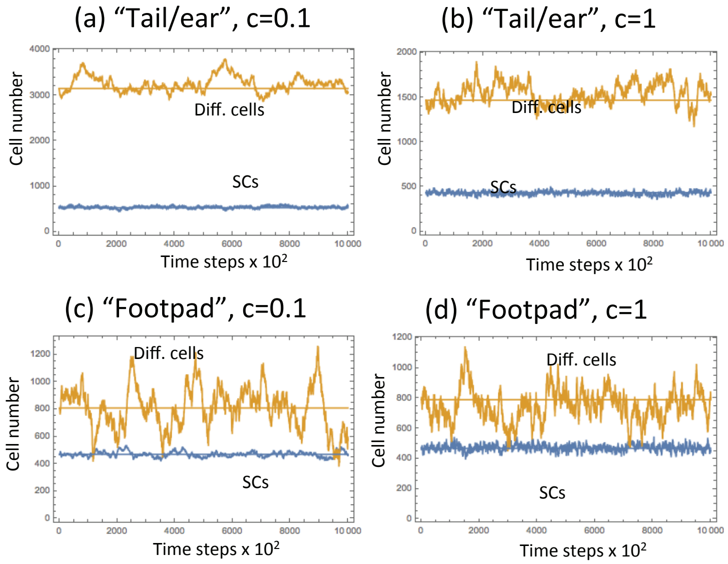

Figure S8: Models combining the effects of hair follicles and micro-injuries. The numbers of differentiated and SCs are plotted as functions of time, for a typical run, with (a,c) $S = 0$ and (b,d) $S = 1$, for models of (a,b) the tail/ear and (c,d) the footpad epidermis. Equations (11-12) of the main text were used with parameters $h = 0.3$, $\epsilon = 0.05$. In figures marked as "Footpad" we used $E = 0$ (no hair follicles), and micro-injuries were modeled by removing 10% of differentiated cells in 0.01% of randomly chosen updates. Figures marked as "Tail/ear" contain no micro-injuries, and the hair follicles were included in the form of the nonzero E term, equation (12) of the main text.

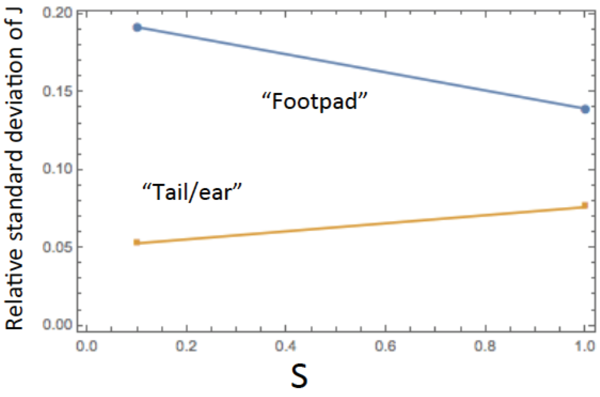

Figure S9: The role of hair follicles and micro-injuries in epidermal turnover. The relative standard deviation of the number of differentiated cells ($\sqrt{Var(J)}/E(J)$) is plotted against the fraction of symmetric divisions, in the models for "Tail/ear" and "Footpad". Parameters are as in figure S8.

death (that is, if we assume that micro- injuries do not remove SCs directly), we have $E = A\eta = 0$ in the footpad. In this case, the steady state level of the differentiated cells is independent of S, and optimization for division symmetry can happen over a different criterion, for example, to minimize the variance. Again, in this case a larger proportion of symmetric divisions is advantageous, as argued in the main text. A numerical example of a system with and without hair follicles is shown in figures 11 and 12 of the main text.

6.2 Combining the effects of the hair follicles and micro-injuries

In the main text the two factors, the hair follicles and the micro-injuries, are analyzed separately. It is easy to combine them. Let us compare the footpad epidermis model, where no hair follicles are present and an increased level of micro-injuries is observed, with the ear/tail epidermis model, where hair follicles serve as an external source of SCs and micro-injuries are insignificant. Figure S8 shows typical trajectories for a model of the tail/ear (in the presence of hair follicles, and no micro-injuries), as wells a model of the footpad (no hair follicles, in the present of micro-injuries). Figure S9 summarizes the results. For the tail/ear model, the relative standard deviation is minimized by asymmetric divisions, and for the footpad model, it is minimized

by symmetric divisions.

Regardless of S, the relative size of fluctuations in systems with hair follicles is lower due to the increase in the overall population size, which in turn is a consequence of the exogenous SC input. A decrease in the steady state population of differentiated cells is also observed in the presence of micro-injuries (modeled as a SC death or differentiated cell death). A reduction in the steady state level due to the absence of an extra source ($E = 0$) and an additional death terms ($A > 0$ and/or $B > 0$) are mathematically necessary. This however appears to be in contradiction with the observation that in the mouse footpad epidermis, the differentiated cell layer is thicker than that in the ear/tail epidermis. We therefore must assume the presence of other factors, unrelated to the SC division symmetries, that increase the thickness of the epidermis in the footpad to compensate for the lack of hair follicles and an increased death rate. These mechanisms remain beyond the framework developed in this paper.

References

Komarova, N. L. (2013). Principles of regulation of self-renewing cell lineages. *PloS one* **8**(9), e72847.

Yang, J., Sun, Z., and Komarova, N. L. (2015). Analysis of stochastic stem cell models with control. *Mathematical biosciences* **266**, 93–107.
